# Linking morphological and molecular sources to disentangle the case of *Xylodon australis*

**DOI:** 10.1038/s41598-020-78399-8

**Published:** 2020-12-15

**Authors:** Javier Fernández-López, M. Teresa Telleria, Margarita Dueñas, Mara Laguna-Castro, Klaus Schliep, María P. Martín

**Affiliations:** 1 Department of Mycology, Real Jardín Botánico-CSIC, Plaza de Murillo 2, 28014 Madrid, Spain; 2 Graz University of Technology, Graz, Austria; 3 Instituto de Investigación en Recursos Cinegéticos, IREC (UCLM-CSIC-JCCM), Ciudad Real, Spain; 4 Centro de Astrobiología (INTA-CSIC), Instituto Nacional de Técnica Aeroespacial Esteban Terradas, Torrejón de Ardoz, Spain

**Keywords:** Molecular evolution, Phylogenetics, Speciation, Taxonomy

## Abstract

The use of different sources of evidence has been recommended in order to conduct species delimitation analyses to solve taxonomic issues. In this study, we use a maximum likelihood framework to combine morphological and molecular traits to study the case of *Xylodon australis* (Hymenochaetales, Basidiomycota) using the *locate.yeti* function from the *phytools* R package. *Xylodon australis* has been considered a single species distributed across Australia, New Zealand and Patagonia. Multi-locus phylogenetic analyses were conducted to unmask the actual diversity under *X*. *australis* as well as the kinship relations respect their relatives. To assess the taxonomic position of each clade, *locate.yeti* function was used to locate in a molecular phylogeny the *X*. *australis* type material for which no molecular data was available using morphological continuous traits. Two different species were distinguished under the *X*. *australis* name, one from Australia–New Zealand and other from Patagonia. In addition, a close relationship with *Xylodon lenis*, a species from the South East of Asia, was confirmed for the Patagonian clade. We discuss the implications of our results for the biogeographical history of this genus and we evaluate the potential of this method to be used with historical collections for which molecular data is not available.

## Introduction

Only six years before the famous wreck of the HMS *Erebus* and HMS *Terror* during Franklin's lost Arctic expedition, Sir James Clark Ross commanded the same two vessels during his Antarctic mission with the purpose of investigating terrestrial magnetism between 1839 and 1843. Onboard the HMS *Erebus* was the British botanist Sir Joseph Dalton Hooker, enrolled as assistant ship's surgeon and to collect natural history specimens. During four years they explored many austral areas from New Zealand and Tasmania to the Antarctic and Tierra de Fuego, including numerous islands, such as Crozet, Kerguelen or Falkland, collecting samples from species never described before. Most of the plant and fungal specimens from this expedition were deposited in the Kew Herbarium and many new fungus species were described by Miles Joseph Berkeley in Hooker’s work “*The Botany of the Antarctic Voyage*”^1^.

Among the new species described by Berkeley, *Grandinia australis* Berk. [≡*Xylodon australis* (Berk.) Hjortstam & Ryvarden], was collected from Tasmania Island. This species is a white rot corticioid fungus described as “*entirely effused and resupinate, without any evident margin, pale, white within, cracked. Hymenium rough with unequal granules, each of which has one or more distinct papellae*”^1^ and characterized by its chestnut-orange hymenial surface that turns violet upon the application of KOH^2^. This longtime neglected species was only known from Australia^2^, but in recent decades, Greslebin et al.^3^ reported it from Argentina and New Zealand, extending its known distribution. Though they found morphological differences in basidiospores among samples from different areas, they maintained the specimens from New Zealand, Australia and Argentina as a single species with an Austral distribution.

In recent decades, there has been a shift in the criteria to identify fungal species. The phylogenetic species recognition (PSR), based on the analyses of DNA sequences, has shown a closer match to an evolutionary species concept than other methods such as the traditional morphological species recognition (MSR) or the biological species recognition, (BSR)^4^. As a result, in recent years there has been an increase of new species described based on DNA data. This shift toward the phylogenetic species recognition has been possible due to the development of new tools to obtain and analyze DNA data. Initiatives such as Assembling the Fungal Tree of Life (AFToL)^5^ or those carried out by the Fungal Barcode Consortium^6^ have led to the identification of the best DNA regions for phylogenetic reconstructions or for new fungal species identification.

The revision of old species names and the study of type specimens are necessary in order to ensure the correct taxonomic classification of biodiversity. However, when DNA sequences are used as the key evidence in the study of fungi, the lack of molecular data from type collections can be a problem. Type specimens are often old, dry material deposited in herbaria where DNA can be poorly preserved or treated with fixatives that have damaged it. Although it has been possible to extract and amplify the nuclear ribosomal Internal Transcribed Spacer region: ITS nrDNA, fungal barcode^6^, from very old specimens, i.e. a specimen of *Agaricus cossus* collected in 1794^7^, or another of *Hyphodermella rosae* from 1926^8^, this is an exception. The success in DNA amplification usually decreases with the specimen age, making it difficult to obtain enough high quality DNA to be used in phylogenetic studies^9^. In addition, some herbaria have special policies about type specimens, and destructive sampling to obtain DNA data is not always allowed due to the historical value of those collections^10^. This is the case of *Xylodon australis* type material, collected during the Ross Antarctic Expedition, which is available in the herbarium of the Royal Botanic Gardens Kew (K) for morphological study, but not for destructive sampling.

Since DNA data have emerged as a vital source of information to identify fungal species and to study their diversity, when molecular data of type specimens is missing the reliability of their classification and/or nomenclature may be compromised. However, in many cases, additional morphological data or geographic origin can help to assign type specimens to a specific clade obtained from molecular phylogenetic analyses^11^. In recent years, several methodological approaches have been developed to include fossils or recently extinct taxa in molecular phylogenies using morphological characters^12,13^. These tools are based on modifications of Felsenstein’s approach^14,15^ to estimate phylogeny from continuous characters. Starting with an ultrametric molecular phylogeny for *N*–1 species and a continuous character dataset, these methodologies are able to infer the position of a new taxon not present in the molecular phylogenetic tree, from the measurements of particular phenotypic characters^12^. The same scheme can be applied to locate a type specimen in a molecular phylogenetic tree in order to solve taxonomic issues when DNA sequences are used in the study of fungi or other organisms.

Here, we address the case of the *Xylodon australis* using the methodologies described above. In order to assess the hidden diversity of *X*. *australis*, two-loci phylogenetic analyses were conducted. No molecular data were obtainable from the nomenclatural type specimen, since destructive sampling was not allowed, due to its historical value. Thus, morphological studies were carried out to place the type material into the molecular phylogenetic tree, using a maximum likelihood framework through the *locate.yeti* function from the *phytools* R package to solve the possible taxonomical issues^16^.

## Results

### Phylogenetic analyses

A total of 66 new sequences were generated in this study: 35 sequences for ITS nrDNA region and 31 for nrLSU. Final alignments including sequences from EMBL/GenBank/DDBJ databases contained 110 ITS nrDNA sequences for a dataset length of 724 characters and 87 nrLSU sequences with 987 characters. All new sequences have been deposited in the EMBL/GenBank/DDBJ database and their accession numbers are presented in Table S1.

The identity of all samples named under *X*. *australis* was confirmed by molecular data (see Supplementary Figs. S1–S3 on line) and their phylogenetic position among other *Xylodon* species was according to Riebesehl et al.^17^. The number of constant, variable, uninformative and informative characters under maximum parsimony analyses are indicated in the legend of the Supplementary Figs. S1–S3.

### Statistical tests of morphological characters

ANOVA on basidia and spore morphology was conducted on eight Australian and 12 Patagonian specimens (Table 1).

**Table 1:** Specimens included in the morphological analysis.

Species/specimens	Country	Basidia morphology^a^		Basidiospore morphology^a^		
		L	W	L	W	Q
***Xylodon australis*** **(Berk.) Hjortstam & Ryvarden**						
K 56442 (Holotype)	Australia	29.8	5.1	7.2	5.1	1.41
CANB569566	Australia	28.58	4.75	7.3	4.6	1.59
CANB569567	Australia	32	4.83	6.8	4.4	1.55
CANB569568	Australia	28	4.3	6.3	4.2	1.50
CANB569570	Australia	26.5	4.75	6.7	4.3	1.56
CANB569572	Australia	30	4.7	7.3	4.4	1.66
CANB751963	Australia	28.7	4.97	6.4	4.2	1.52
CANB752080	Australia	29.65	4.5	7	4	1.75
CANB752088	Australia	28.3	4.7	6.7	4.1	1.63
CANB869100	Australia	28.85	5	6.5	4	1.63
CANB869124	Australia	30.3	5	6.3	4.1	1.54
PDD 23689	New Zealand	28.5	4	7.1	4.6	1.54
PDD 23691	New Zealand	30.56	5	6.8	5.2	1.31
PDD 23692	New Zealand	29.75	4.85	6.85	5.2	1.32
PDD 23693	New Zealand	31	4.75	6.8	4.9	1.39
PDD 23694	New Zealand	29.8	4.5	6.6	4.6	1.43
PDD 23696	New Zealand	30.45	4.67	6.1	4	1.53
PDD 23698	New Zealand	30.66	4.33	6.6	4.5	1.47
PDD 23699	New Zealand	26.33	4.66	6.7	4.4	1.52
PDD 23703	New Zealand	25.83	4	6.9	5	1.38
PDD 23704	New Zealand	29.5	4	6.4	4.7	1.36
PDD 23705	New Zealand	30.1	4.7	6.2	4.4	1.41
***Xylodon lenis*** **Hjortstam & Ryvarden**						
Wu 890714 (Isotype)	Taiwan	18.5	3.75	4.75	3.25	1.46
***Xylodon magallanesii*** **sp. nov.**						
AG 730	Argentina	22.65	4	5.6	3	1.87
AG 1548	Argentina	23.75	4.62	6.1	2.9	2.10
AG 1872	Argentina	23.22	4.35	6	3	2.00
MA-Fungi 90397, 20008 Tell. (holotype)	Chile	24.5	4.75	5.6	2.87	1.95
MA-Fungi 90391, 14120 MD	Chile	21.4	4	6.4	3	2.13
MA-Fungi 90392, 14163 MD	Chile	23	4	5.8	3	1.93
MA-Fungi 90393, 14164 MD	Chile	22.5	4.5	6	3	2.00
MA-Fungi 91815, 14629MD	Chile	22.5	4.25	5.54	2.45	2.26
MA-Fungi 91816, 15630MD	Chile	27	4	5.1	2.65	1.92
MA-Fungi 91817, 15632MD	Chile	23.3	3.83	5.95	2.77	2.14
MA-Fungi 91818, 15634MD	Chile	–	–	5.17	2.50	2.06
MA-Fungi 91819, 15637MD	Chile	24	4.16	5.6	2.45	2.28
MA-Fungi 91820, 15638MD	Chile	–	–	–	–	–
MA-Fungi 91821, 20007Tell	Chile	23	4.5	5.72	2.90	1.97
PDD 69093, MR 11041	Argentina	21.85	4	6.3	3	2.10

L = length, W = width, Q = length/width ratios.

^a^(–) = Not observed.

**Figure 1:**
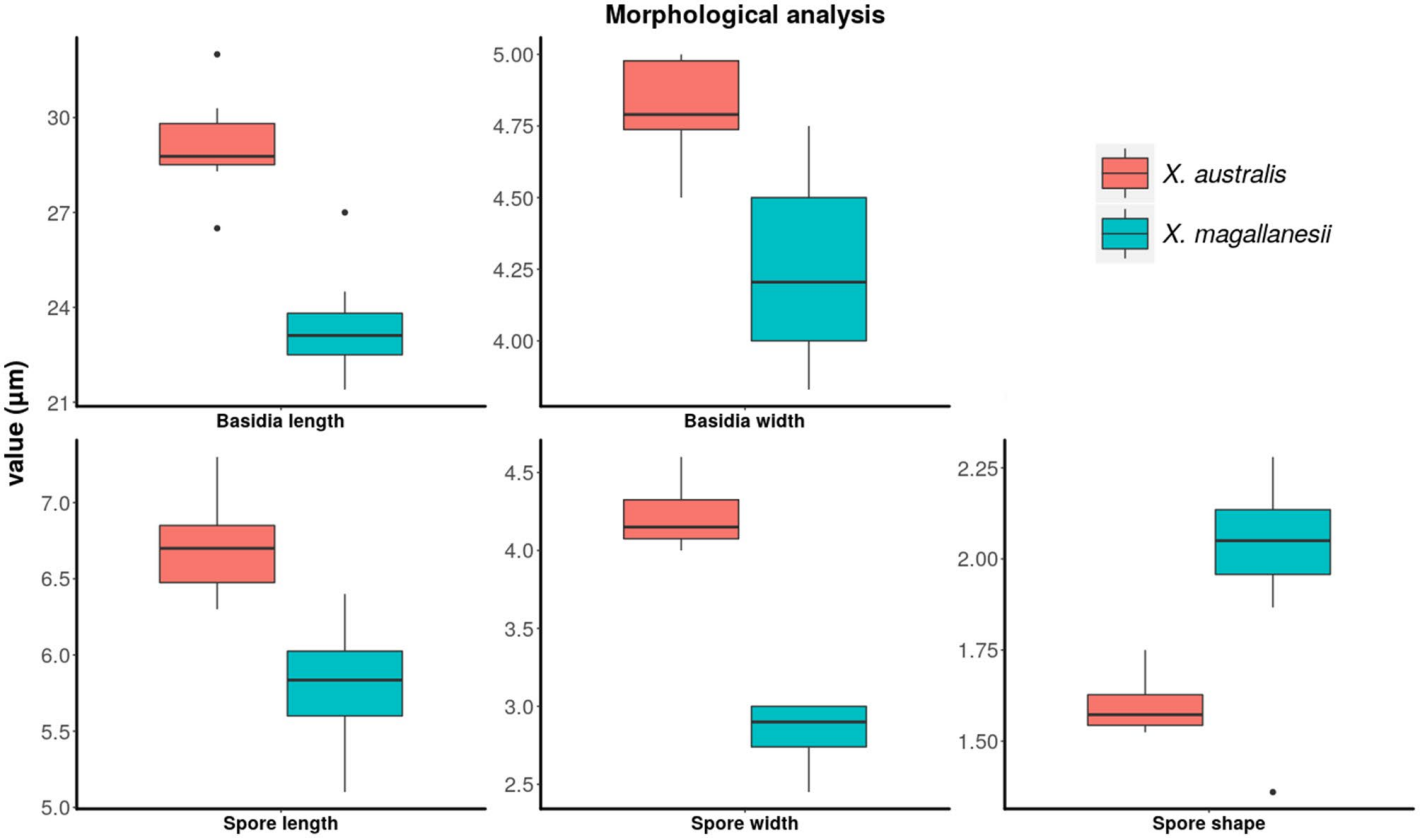
Morphological analyses conducted for basidia length and width, and spore length, width and length/width relation. Graphs were generated using R Core Team v3.6.1 (https://www.R-project.org/).

Significant differences were found for all measures: basidia length and width, and spore length, width and length/width relation (Table 2; Fig. 1). Specimens from Australia showed longer and wider basidia than samples from Patagonia. In the same way, spores of specimens from Australia were longer and wider than those of Patagonian samples. Spore length/width ratio for the Australian lineage was lower than for the Patagonian clade, thus the former has spores narrowly ellipsoid or subcylindric compared to the latter.

**Table 2:** Statistical tests of morphological characters.

	F(1, 18)	P-value
Basidia length	70.34	< 0.01
Basdia width	23.65	< 0.01
Spore length	30.50	< 0.01
Spore width	208.4	< 0.01
Spore length/width	22.13	< 0.01

ANOVA on basidia and spore morphology.

### Inferring the position of the nomenclatural type of *Xylodon australis* in the molecular phylogenetic tree

**Figure 2:**
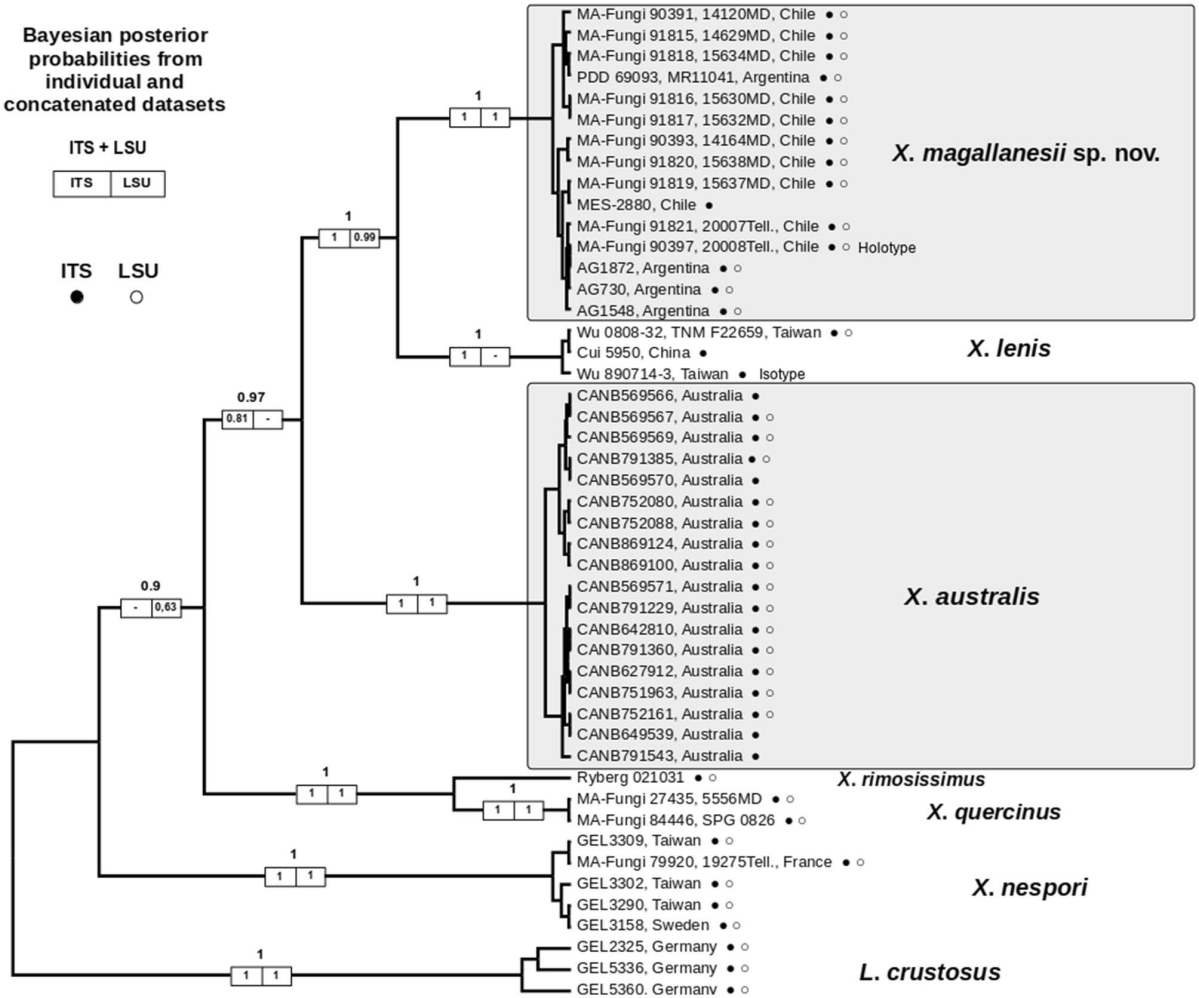
Ultrametric phylogenetic trees to obtain the *Xylodon australis* crown group. Topology showed correspond to the bayesian tree for combined ITS nrDNA + nrLSU datasets. The tree was edited using FigTree v1.4.4 (http://tree.bio.ed.ac.uk/software/figtree/).

The ultrametric phylogenetic trees of 47 *Xylodon* specimens gave the same topology for the ITS nrDNA (not shown), nrLSU (not shown), and ITS nrDNA + nrLSU datasets (Fig. 2). Effective sample sizes for all parameters were higher than 200. Bayesian inference analyses showed that specimens under the *Xylodon australis* name were distributed in two non-directly-related and highly supported clades. All Australian collections were grouped in one clade, while the other clade included all the Chilean and Argentinean specimens. Three sequences of species *Xylodon lenis* (including from the type specimen) are the sister clade of the Chilean and Argentinean specimens; this relationship has strong support in all datasets (ITS nrDNA PP = 1.0, nrLSU PP = 0.99, and ITS nrDNA + nrLSU PP = 1). *Xylodon lenis*, *X. australis* from Australia and *Xylodon gr. australis* from Patagonia formed the crown clade for all *X. australis* specimens (ITS nrDNA + nrLSU PP = 0.97).

**Figure 3:**
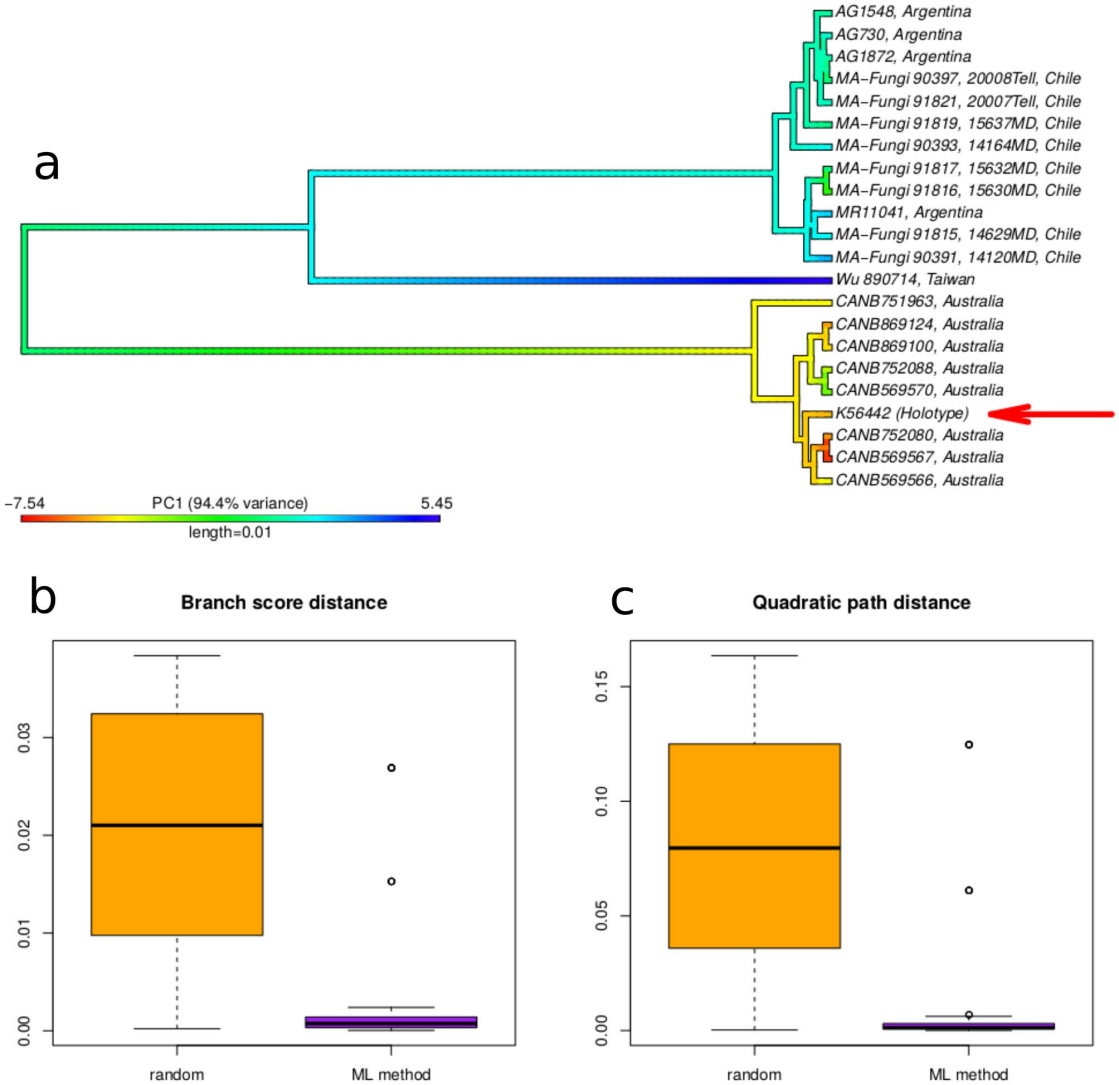
Results of analysis to infer the position of the type material of *Xylodon australis* using *locate.yeti* function. (**a**) Position of the type material. (**b**,**c**) Branch score and quadratic paths distances from the original molecular tree. All plots were generated using R Core Team v3.6.1 (https://www.R-project.org/).

The topology of the combined dataset for this clade was used as subtree to infer the phylogenetic position of *Xylodon australis* type specimen using continuous morphological traits (Fig. 3a), as well as to the New Zealand samples from which no sequences were obtained (see Supplementary Fig. S4).

The type specimen of *Xylodon australis* was located in the Australian molecular lineage, according to the five continuous morphological traits used, under a maximum likelihood framework using the *locate.yeti* (Fig. 3a).

Results of the randomization test to assess the accuracy of the method for our case study are shown in Fig. 3b,c. In general, trees reconstructed according to continuous mophological traits, that is, using *locate.yeti*, were more similar to the actual molecular tree than those reconstructed by random tip location (lower values of branch score and quadratic paths distances, Fig. 3b,c). The likelihood ratio test was conducted by constraining the type position to the Patagonian clade (the alternative position to our results). The hypothesis that the type collection of *Xylodon australis* belongs in the Patagonian clade was rejected by our analyses (P-value < 0.01; Table 3).

**Table 3:** Likelihood ratio test conducted for constrained and unconstrained type position to the Patagonian clade.

Model	log(L)	P-value (compared to unconstrained model)
Unconstrained	− 14.27	–
Constrained to Patagonian clade	− 35.24	< 0.01

## Taxonomy

**Figure 4:**
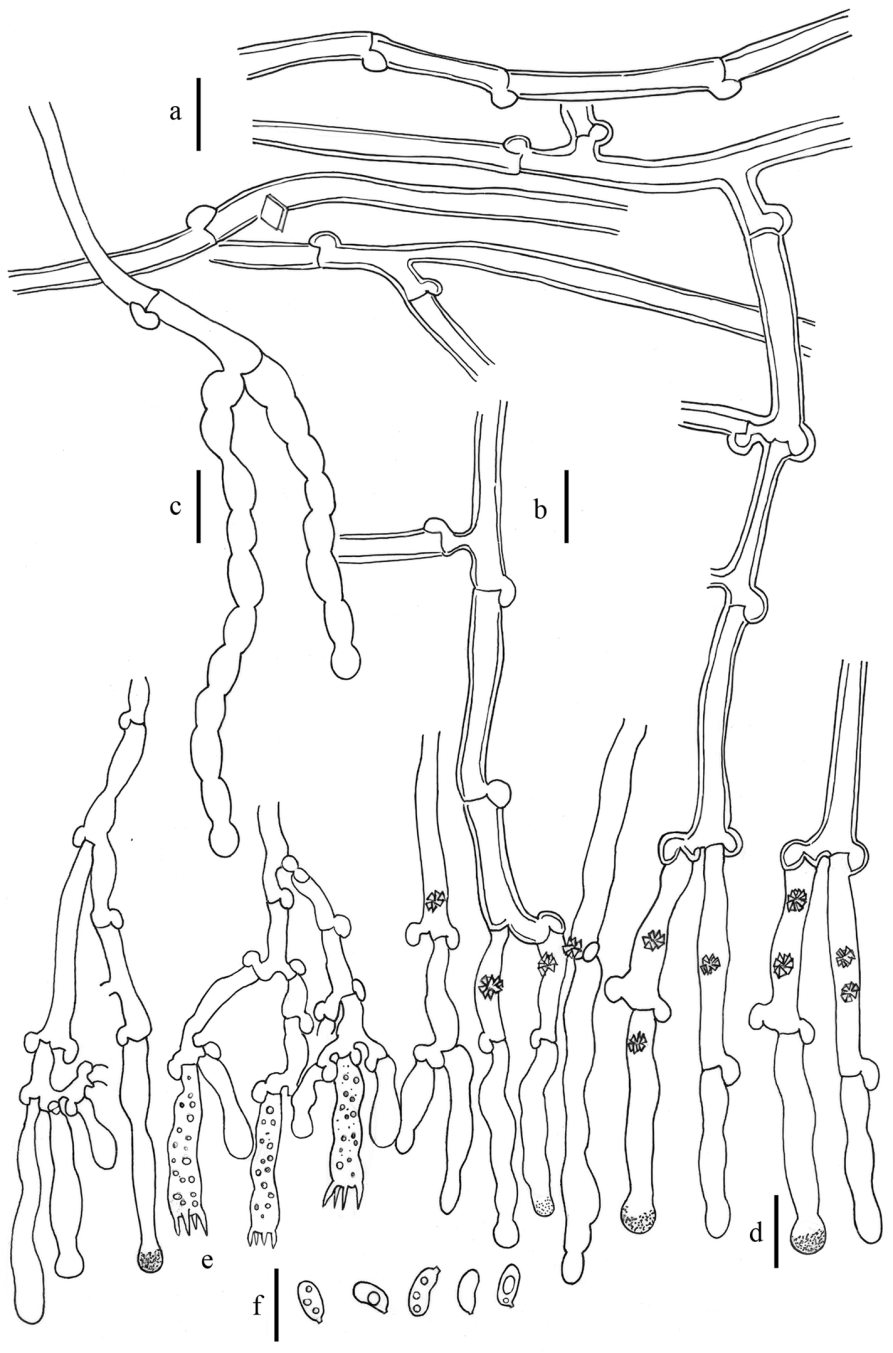
*Xylodon magallanesii*, 20008Tell, MA-Fungi 90397, holotype. (**a**) Subicular hypha. (**b**) Subhymenial hypha. (**c**) Moniliform cystidia. (**d**) Claviform cystidia. (**e**) Basidia. (**f**) Basidiospores. Bar = 10 µm. Hand-made draws were edited using GIMP v2.10.20 (https://www.gimp.org/).

**Figure 5:**
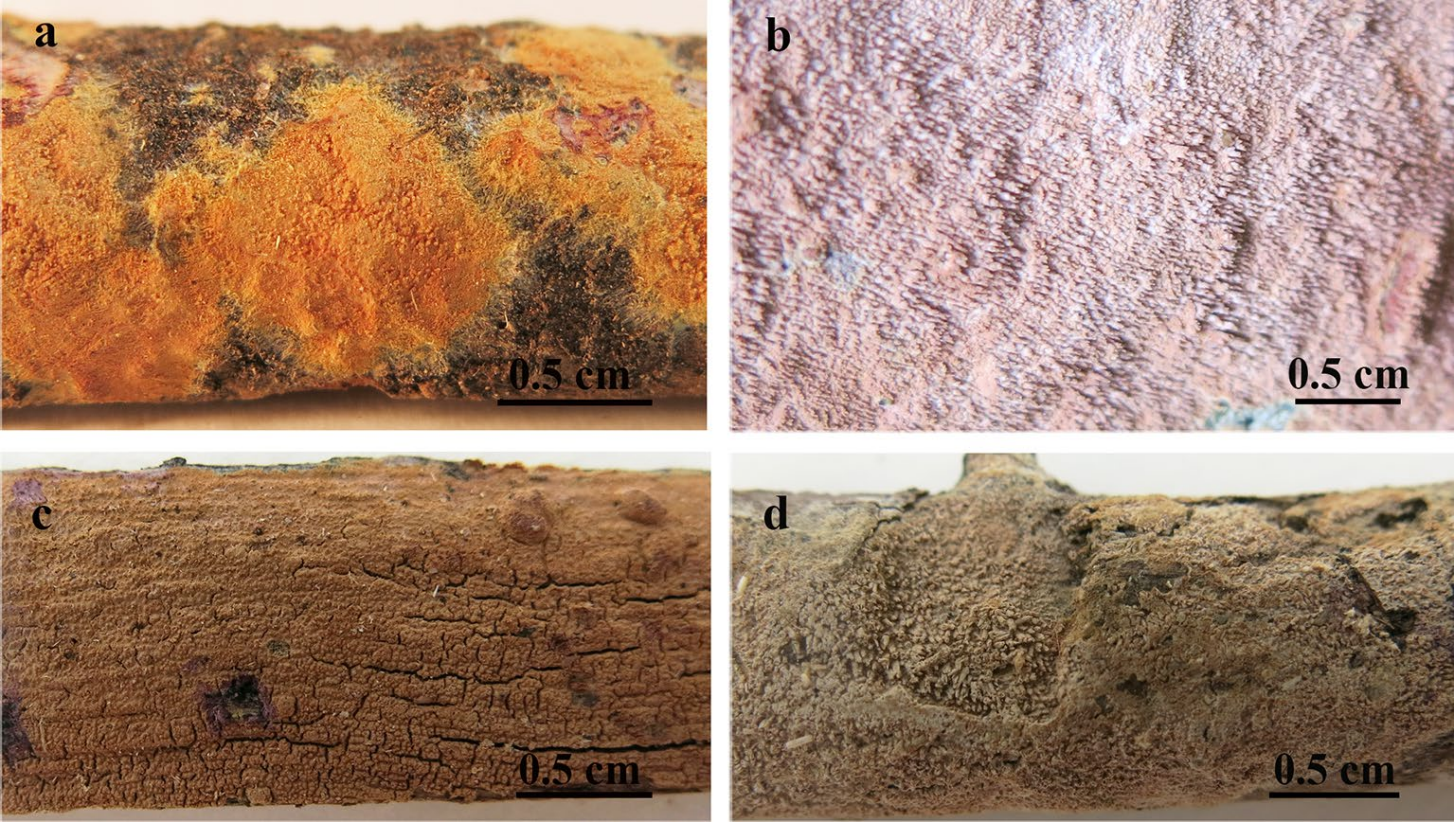
*Xylodon magallanesii*, (**a**) 20008Tell, MA-Fungi 90397, holotype, basidiome, dry specimen; (**b**) 15637MD, MA-Fungi 91819, basidiome, wet specimen. *Xylodon australis*, (**c**) P. Wellman 697, CANB 869100, basidiome, dry specimen. *Xylodon lenis*, (**d**) SH Wu 890714-3, H 7027389, isotype, basidiome, dry specimen.

*Xylodon magallanesii* J. Fernández-López, Telleria, M. Dueñas, M. Laguna & M.P. Martín, *sp. nov.* Figures 4, 5a,b (MycoBank MB 834687).

Etymology: Named after Fernando de Magallanes (1480–1521), the Portuguese explorer who commanded the Spanish expedition to the East Indies from 1519 to 1522, resulting in the first circumnavigation of the Earth, which was completed by Juan Sebastián Elcano.

Type: CHILE: Los Lagos (X Región), Palena, Comuna Hualaihué, Reserva de Huinay, road to Lloncochaigua river, 42º22′38.9″S 72º24′45.8″W, 190 msl, on angiosperm dead wood, 30 Apr. 2012, *M. Dueñas, M.P. Martín & M.T. Telleria*, *20008Tell.* (holotype MA-Fungi 90397). ITS nrDNA and nrLSU sequences: GenBank MT158729 and MT158765.

Diagnosis: Morphology similar to *Xylodon australis*, but differs in having smaller basidia, 24–21 × 4–4.5 µm, and smaller and narrowly ellipsoidal to subcylindrical basidiospores, (5–)5.5–6(–6.5) × 2.5–3(–3.5) µm with Q = 2.03.

Basidioma resupinate, effuse; hymenophore sometimes cracked, odontoid or hydnoid, with unequal granules or teeth, 1–4/mm, light pink to dark pink for wet material (4.l.Pink–5.m.Pink–6.d.Pink), light orange to deep orange for dry material (52.l.O–50.s.O–51.deepO), violet in KOH; margin not clearly differentiated. Hyphal system monomitic; generative hyphae hyaline, thin to thick-walled, with clamps, 3–5 µm in diam.; subicular hyphae interwoven, walls up to 1 µm thick, scarcely branched. Subhymenial hyphae thin, branched. Cystidia present: (1) moniliform cystidia scarce, arise from the subiculum, 35–45 × 2.5–5 µm, thin-walled, sometimes with basal clamp; (2) claviform to slightly moniliform cystidia, sometimes with a granulose cap in the hymenium, 25–35 (– 45) × 3–5 µm, thin-walled, basal clamp. Basidia narrowly clavate, 21–24 × 4–4.5 µm, four sterigmata, with basal clamp. Basidiospores narrowly ellipsoidal to subcylindrical, (5–)5.5–6(–6.5) × 2.5–3(–3.5) µm, hyaline, thin-walled, smooth, usually with several oil drops. L = 6.06, W = 2.98, Q = 2.03 (n = 20).

Hosts & Habitat: On dead wood of *Nothofagus betuloides*, *N*. *dombeyi*, *N*. *pumilio*, *Amomyrtus luma* and *Drymis winteri.*

Known distribution: Reported from the Patagonian region (southern Chile and southern Argentina).

Additional specimens (paratypes) examined: ARGENTINA: Río Negro, Parque Nacional Nahuel Huapi, Puerto Blest, road to Los Cántaros, on fallen log of *Nothofagus dombeyi*, 31 October 1995, *M. Rajchenberg 11041* (PDD 69093); Tierra de Fuego, Ushuaia, Paso Garibaldi, on *Nothofagus pumilio* or *N. betuloides*, 27 Mar. 1998, *A. Greslebin 1548*; ibid., *Nothofagus betuloides*, 10 November 1998, *A. Greslebin 1872*; Tierra de Fuego, Ushuaia, Tolhuin, 3 km East from Hostería Kaikén, on *Nothofagus pumilio*, 4 November 1996, *A. Greslebin 730*. CHILE: Los Lagos (X Región), Palena, Comuna Hualaihué, Reserva de Huinay, “cementerio de los alerces”, 42º21′57.9″S 72º24′56.9″W, 30 msl, on *Amomyrtus luma*, 29 April 2012, *M. Dueñas, M.P. Martín & M.T. Telleria*, *14120MD* (MA-Fungi 90391); ibid., “Derrumbe Antiguo”, 42º22′17.0″S 72º24′12.2″W, 120 msl, on *Nothofagus dombeyi*, 1 May 2012, *M. Dueñas, M.P. Martín & M.T. Telleria*, *14163MD* (MA-Fungi 90392); idem, *14164MD* (MA-Fungi 90393); ibid., 42º22′01.5″S 72º24′57.8″W, 50 msl, on *Drymis winteri*, 10 May 2013, *M. Dueñas, M.P. Martín & M.T. Telleria*, *14629MD* (MA-Fungi 91815); ibid., road to Lloncochaigua river, 42º22′38.9″S 72º24′45.8″W, 190 msl, on dead wood, 4 May 2013, *M. Dueñas, M.P. Martín & M.T. Telleria*, *20007Tell.* (MA-Fungi 91821); Los Ríos (XIV Región), Ranco, Comuna de La Unión, road T-80, 40º13′49.3″S 73º21′38.4″W, 664 msl, on dead wood, 6 November 2017, *M. Dueñas, J. Fernández-López, M.P. Martín, S. Nogal-Prata & M.T. Telleria*, *15630MD* (MA-Fungi 91816); idem, *15632MD* (MA-Fungi 91817); idem, *15634MD* (MA-Fungi 91818); idem, *15637MD* (MA-Fungi 91819); idem, *15638MD* (MA-Fungi 91820).

**Figure 6:**
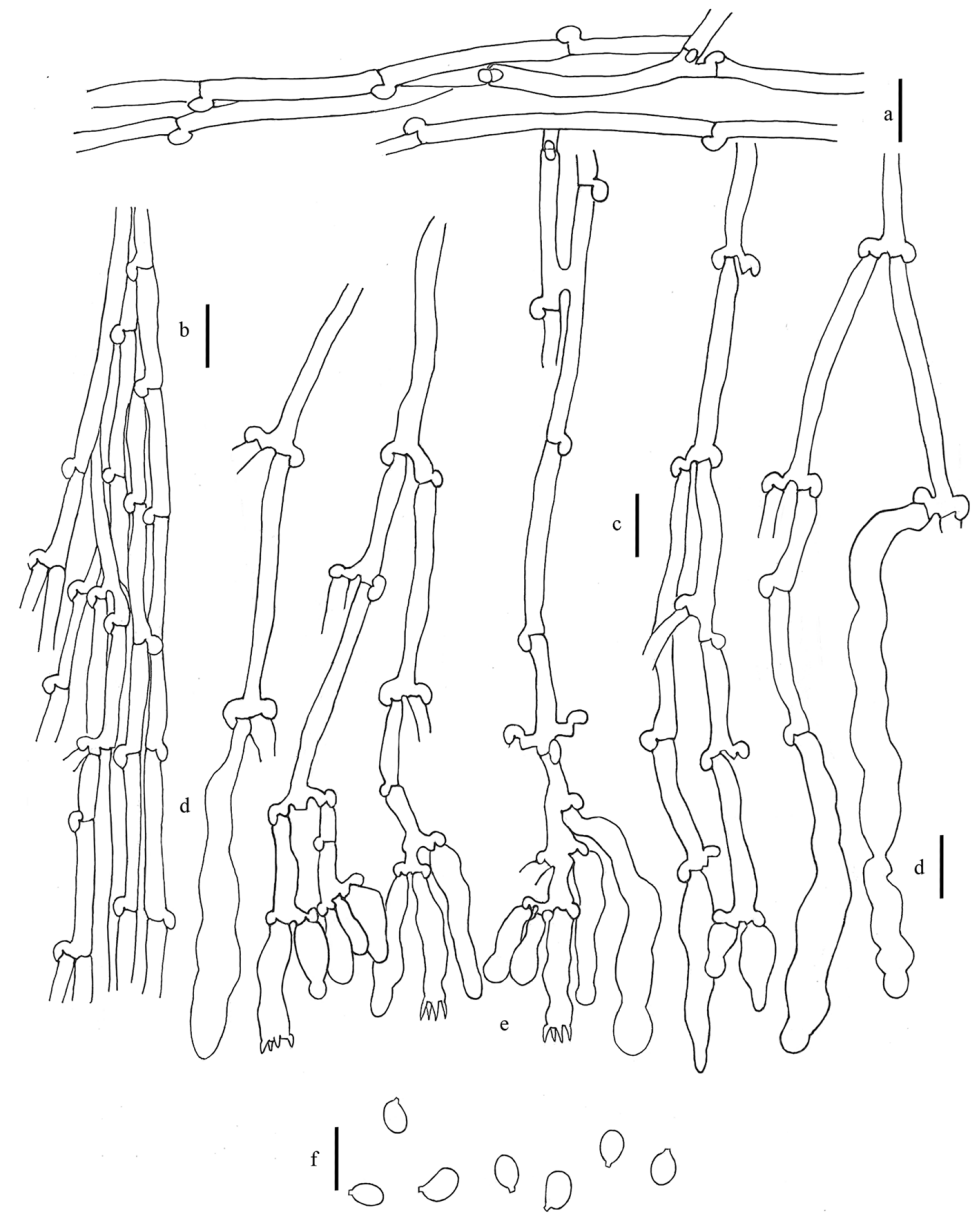
*Xylodon lenis*, SH Wu 890714-3, H 7027389, isotype. (**a**) Subicular hypha. (**b**) Hypha of aculeus. (**c**) Subhymenial hypha. (**d**) Cystidia. (**e**) Basidia. (**f**) Basidiospores. Bar = 10 µm. Hand-made draws were edited using GIMP v2.10.20 (https://www.gimp.org/).

Other material examined (*Xylodon australis*, Figs. 5c, 6): AUSTRALIA: Australian Capital Territory: “Birrigai”, 22 km SW of Capital Hill, Canberra, 35º28′S 148º57′E, 700 msl, in an open paddock, 16 May 1992, *H. Lepp 818* (CANB 569566); ibid., Orroral to Cotter Hut road, 38 km SW of Capital Hill, Canberra, 35º37′S 148º55′E, 1100 msl, on fibrous bark of live *Eucalyptus* trunk, 12 Juny 1993, *H. Lepp 964* (CANB 569567); ibid., Tidbinbilla Nature Reserve, 27 km SW of Capital Hill, Canberra, 35º27′S 148º53′E, 800 msl, on a fallen, rotting *Eucalyptus* trunk, 21 February 1993, *H. Lepp 905* (CANB 569570); New South Wales, Southern Tablelands, Morton National Park, near Endrich River, Round mountain, *Eucalyptus* forest, 35º10′12″S 150º09′37″E, 700 msl, on small branch on ground, 26 July 2011, *P. Wellman 697* (CANB 869100); ibid., Brindabella National Park, near Canberra. Doctors Flat Road, open *Eucalyptus* forest, 35º13′48″S 148º52′40″E, 887 msl, on *Eucalyptus* log, 20 December 2011, *P. Wellman 711A* (CANB 869124); Queensland, Darling Downs, Girraween National Park, *Eucalyptus* dominated woodland, 3 May 2005 (CANB 751963); ibid., Burnett, Bunya Mountains National Park, rain forest, 6 May 2005 (CANB 752080); ibid., a little below the summit of Mt Kiangarow, 26º49′45″S 151º 33′00″E, 1130 msl, on rotting branch of live tree, 6 May 2005, *H. Lepp 4827* (CANB 752088). TASMANIA: unlocalized, ex herb M.J. Berkeley (type K(M) 56442); Flowery Gully, on the underside of a rotting log, 21 April 1992 (CANB 569568); ibid., Gunner's Quoin, Hobart, *Eucalyptus* woodland 28 April 1992 (CANB 569572). NEW ZEALAND: Auckland, Mt. Te Aroha, 600 msl, on *Brachyglottis repanda*, November 1946, *G.H. Cunningham* (PDD 23689); ibid., 900 msl, on *Coriaria arborea*, November 1946, *G.H. Cunningham* (PDD 23691); ibid., Thames, Waiomo Valley, on *Coriaria arborea*, 21 August 1954, *J.M. Dingley* (PDD 23692); ibid., Camel’s Back, Coromandel, 800 msl, on *Coriaria arborea*, 25 October 1954, J.M. Dingley (PDD 23693); ibid., Little Huia, 200 msl, on *Leptospermum ericoides*, 24 December 1949, *E.E. Chamberlain* (PDD 23696); ibid., Swanson, on *Leptospermum ericoides*, 18 April 1954, *J.M. Dingley* (PDD 23698); ibid., Whakarewarewa, Rotorua, on *Eucalyptus globulus*, 14 Juny 1950, *J.M. Dingley* (PDD 23699); ibid., Waipous Kauri Forest, on *Leptospermum ericoides*, 30 September 1949, *J.M. Dingley* (PDD 23703); ibid., Huia, on *Leptospermum ericoides*, 17 Jannuary 1955, Mrs. E.E. Chamberlain (PDD 23704); ibid., Piha, Glen Esh Valley, on *Leptospermum ericoides*, 31 March 1956, *J.M. Dingley* (PDD 23705); Westland, Fox Glacier Road, 600 msl, on *Coriaria arborea*, November 1946, *G.H. Cunningham* (PDD 23694).

**Figure 7:**
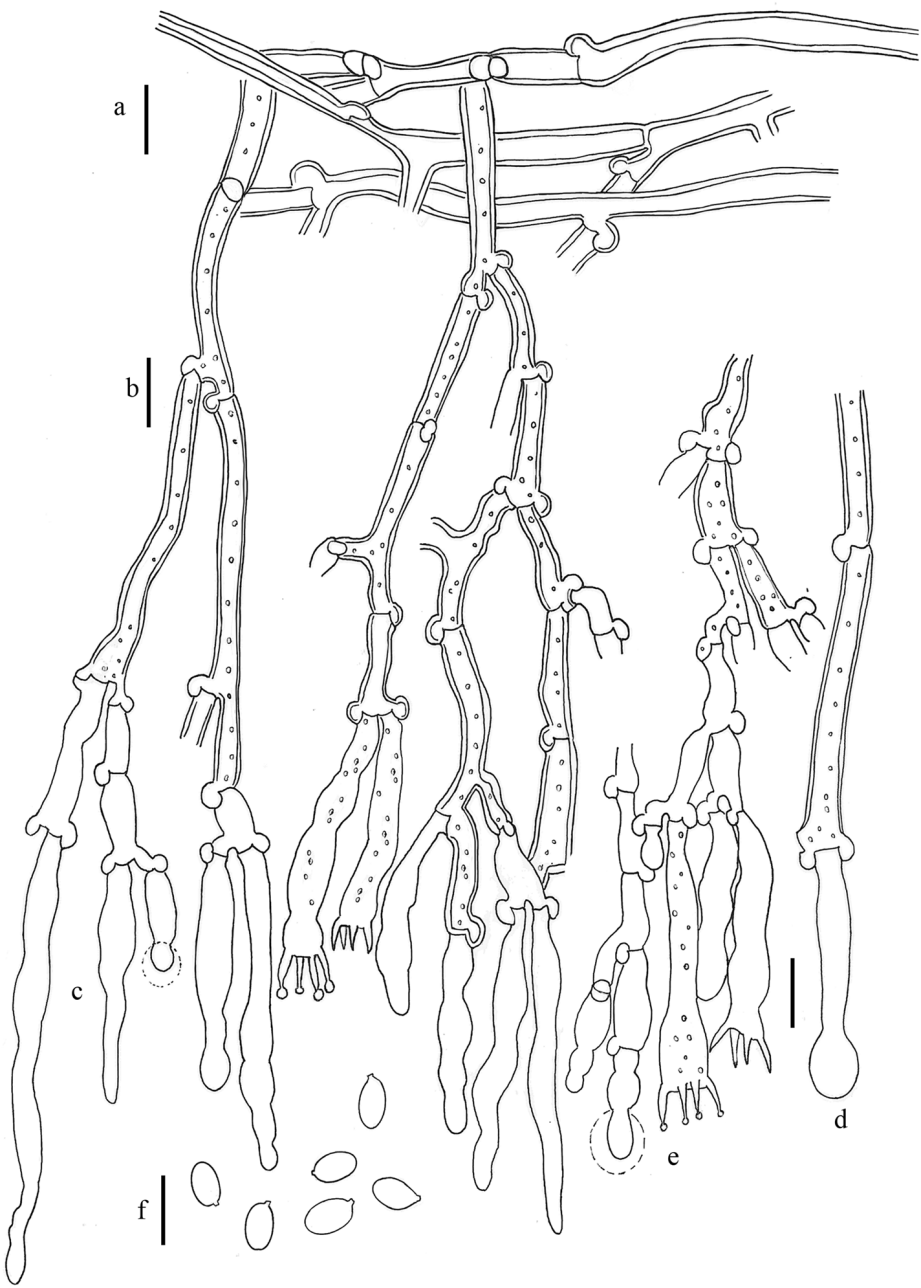
*Xylodon australis*, CANB 869100. (**a**) Subicular hypha. (**b**) Subhymenial hypha. (**c**) Subulate cystidia. (**d**) Capitate cystidia. (**e**) Basidia. (**f**) Basidiospores. Bar = 10 µm. Hand-made draws were edited using GIMP v2.10.20 (https://www.gimp.org/).

Notes: Although, according the molecular analysis based on ITS nrDNA and nrLSU sequences, *Xylodon magallanesii* is closely related to *Xylodon lenis* (Fig. 2), it is macromorphologically closer to *Xylodon australis* (Fig. 7), having both odontoid to hydnoid hymenophore, with flat and scattered theeth. In addition to their smaller basidia (21–24 × 4–4.5 µm in *X*. *magallanesii* and 21–33 × 3.5–5 μm in *X*. *australis*) and basidiospores (5.5–6 × 2.5–3 µm in *X*. *magallanesii* and 6–7.5 × 3.5–5 μm in *X*. *australis*), *X. magallanesii* can be distinguished from *X*. *australis* by their smaller claviform cystidia with a granulose cap, up to 45 µm length. There are clear morphological differences between *X*. *lenis* and *X*. *magallanesii*. *Xylodon lenis* have hydnoid hymenophore with conical to subcylindrical aculei, presents smaller basidia (from 16 up to 21 µm) and smaller and broadly ellipsoidal spores (4.2–5 × 3–3.5 μm). A shared character among the three species is the hymenophore color change after the application of KOH, turning from orange to violet.

Other material examined (*Xylodon lenis*, Figs. 5d, 7): TAIWAN: Kaohsiung, Liukuei Hsiang, Shanping, 770 msl, on fallen twig of angiosperm, 14 July 1989, *Sheng Hua Wu 890714-3* (isotype).

## Discussion

The exhaustive study carried out by Gresbelin et al.^3^ was not enough to consider *Xylodon australis* and *Xylodon magallanesii* as two different species due to the lack of evidence in addition to morphology. However, these authors pointed toward a speciation process due to the differences found in spore size and shape of samples from each area. Our phylogenetic analyses not only confirmed the identity of two species under the *X*. *australis* name, but also revealed the relation of *Xylodon lenis* as the sister species of *X. magallanesii* (Fig. 2).

Our microscopic studies agree with Gresbelin et al.^3^, and showed statistically significant differences in basidia and spore size and shape between *Xylodon australis* and *Xylodon magallanesii* (Fig. 1). Spores of *X*. *magallanesii* were in general smaller, as were their basidia. This correlation between spore and basidia size has been traditionally reported^18,19^ and, therefore, our results could be expected. However, spore shape can be related to more complex responses, including dispersal abilities, bioclimatic fitness, or life history characteristics^11,20^, so a specific study should be carried out to explain differences in basidiospore shape between *X*. *australis* and *X*. *magallanesii*. No molecular data were obtainable from New Zealand samples identified under the *X*. *australis* name, probably due to problems in material conservation (M. Padamsee, PDD Fungarium Curator, pers. comm.); for this reason, these specimens were not included in our statistical analysis in order to compare only those clades confirmed by molecular data. Taking into account our results from *X*. *magallanesii*, and despite the fact that our ML analyses using the *locate.yeti* function included all New Zealand samples in the Australian clade, future analyses should be carried out in order to confirm the identity of the *X*. *australis* samples from New Zealand. In contrast to the type specimen of *X*. *australis*, samples from PDD addressed in this study are available for destructive sampling, and therefore new approaches for DNA extraction could be successful.

The inferred position by *locate.yeti* function for the type *of Xylodon australis,* in the molecular tree arranged with Australian samples, supported the designation of *Xylodon magallanesii* samples from Chile and Argentina as the new species (Fig. 3a). Our analysis resulted in a high performance in the validation test, and was able to correctly locate the pruned molecular taxon better than randomness for our study group (Fig. 3b,c). These results may be due to choosing morphological traits to infer the phylogenetic position, which are known to be taxonomically informative in differentiating closely related species in *Xylodon*^21^. Since the performance of the method is known to increase with the amount of data^11^, the quality of morphological traits could be a key factor when the number of characters is small or the number of taxa in the molecular tree is less than 20, as in our case. In addition, the likelihood ratio test rejected the alternative position proposed for the *Xylodon australis* type in the *X*. *magallanesii* clade. Therefore, its position with the Australian samples is strongly supported. These results are also in accord with geographic evidence, since the type specimen of *X. australis* was collected in Tasmania and therefore their connection with specimens from Australia was expected.

The close phylogenetic relation between *Xylodon australis*, *X*. *lenis* and *X*. *magallanesii* is reported in our study for the first time. *Xylodon lenis* was described as *Hyphodontia mollis* by Wu, in 1990 from Taiwan^22^. Though the *X*. *australis* characteristic color change with the application of KOH was not described for *X*. *lenis*, Wu^22^ highlighted the presence of granular material over the hyphal system that dissolves in KOH, a character also shown by *X*. *australis* and *X*. *magallanesii*. In an additional macromorphological inspection conducted on an *X*. *lenis* isotype (Wu 890714-3) for this study, we could observe color change from orange toward violet after the application of KOH. Therefore, as in other fungal groups^23,24^, this character emerges as a useful trait for taxonomic classification when closely related species are compared. Another morphological character that points to a relation among these three species is the cracked hymenial surface, described in several studies^3,22^, and also shown by *X*. *magallanesii*.

From a biogeographical point of view, these phylogenetic relations are a challenge due to the distribution pattern of the species. The spatial structure inside each species remains congruent and a geographic isolation between them is shown in our results (Fig. 2). The sister relation between *Xylodon magallanesii* from Patagonia and *Xylodon lenis* from continental China and Taiwan could be explained as an example of long distance dispersion^25^. This ability has been confirmed for many fungi, even for the same South America/Asia/Australia pattern in some cases, such as for the *Ganoderma applanatum*-*australe* species complex^26^. Other possible explanations for the disjunct distribution of sister species may be due to incomplete taxon sampling, or to the extinction of lineages that had linked these species in the past.

Epitypification and neotypification have been proposed as possible solutions to address taxonomic confusion in those cases where: type specimens are damaged, characters used for species identification are not manifest, or the material is not available^27^. However, these solutions should be applied cautiously, since they have many other associated risks^27^. Some other authors^10^ have argued that, although the best possible solution would be to examine the type material, one option could be to describe new and well-documented taxa and to ignore old species names, but other problems such as taxonomic inflation, could arise with this practice^28^.

Our study shows how new methodological frameworks can help to solve old taxonomic problems that have become more evident during the DNA era. The possibility to place a type collection into a molecular tree, using phenotypic traits, increases the value of herbaria and museum collections. This is especially important in groups such as *Xylodon*, in which new species and combinations are being proposed every year^19^, and taxonomy is quickly changing^29^. The study of type materials is essential to avoid bad taxonomy that could lead to important ecological and economic losses^30^.

## Material and methods

### Taxon sampling and morphological studies

A total of 37 specimens of *Xylodon australis* from five different herbaria and private collections were analyzed in this study: Australian National Herbarium (CANB), New Zealand Fungal & Plant Disease Collection (PDD), Real Jardín Botánico from Madrid (MA-Fungi), and Alina Greslebin and Mario Rajchenberg private collections (Table 1). In addition, the type specimen of *X*. *australis* (under *Grandinia australis* Berk.) from Royal Botanic Gardens Kew K(M) was also studied morphologically. Basidioma colors were recorded according to Kelly and Judd^31^. Color changes were examined with 3% aqueous KOH. Microscopic measurements were made from sections mounted in aqueous solutions of 3% KOH and 1% aqueous solution of ammoniacal Congo red or 1% aqueous floxine. Sections were examined at magnifications up to 1250× using an Olympus BX51 microscope. Six basidia were measured from each sample. The width (W) and length (L) of 10 spores were also measured and length/width ratios (Q) were calculated. Average values of each character were calculated for each specimen. Additional morphological measurements were performed to provide a general description of each species. Drawings were made with the aid of a drawing tube.

### DNA extraction, amplification and sequencing

Genomic DNA isolation was performed using DNeasy Plant Mini Kit (Qiagen, Valencia, California, USA) following the manufacturer’s instructions, except in three steps: the incubation with the RNAase was done overnight at 65 °C, a second drying at 20,000×*g* was done for 2 min after cleaning with AW buffer, and elution buffer was preheated to 60 °C. When this extraction was not successful, FTA Indicating Micro Cards (Cat Nº WB120211, Whatman, Maidstone, England) were used following the protocol in Telleria et al.^32^. DNA amplifications, purifications and sequencing protocols are deposited in protocols.io (10.7504/protocols.io.wpdfdi6).

Polymerase chain reactions (PCR) were performed to amplify DNA from two loci using the following primer combinations: ITS5/ITS4^33^ were used to obtain DNA amplifications of the nuclear ribosomal internal transcribed spacer regions ITS1 and ITS2, including 5.8S, ITS nrDNA barcode^6^ and LR0R/LR7r for nrLSU region (1–1583)^34,35^. When these pairs of primers failed, both regions were amplified in two parts: in the case of ITS nrDNA, the region ITS1, including part of the 5.8S, with primers ITS5 and ITS2^33^, and part of 5.8S and the region ITS2 with primers ITS3 and ITS4^33^; in the case of nrLSU, one region between the pair of primers LR0R and LR5^33^ and another region between the primers LR3R and LR7r^35^ were amplified. When neither direct nor amplification by parts gave good amplicons (above 20 ng/μL concentration), two semi-nested or nested PCR was used. For ITS nrDNA, a first amplification was done with ITS1F^36^ and ITS4B^36^ primers, amplifying part of the 18S and 28S nuclear ribosomal genes, and a second amplification was done with the pair of primers ITS5/ITS4 (nested-PCR) or only one of the inner primer and one external primer (semi-nested PCR). For nrLSU the first amplification was done with LR0R and LR7r primers, and the second amplifications were done with LR0R/LR5, and LR3R/LR7r primers. Individual reactions to a final volume of 25 μL were carried out using Illustra PureTaq Ready-To-Go PCR Beads (GE Healthcare, Buckinghamshire, UK) with a 10 pmol μL^−1^ primer concentration following the thermal cycling conditions used in Martín and Winka^37^. Negative controls lacking fungal DNA were run for each experiment to check for contamination.

The PCR products were subsequently purified using two different methods. When the quality of the DNA was low, due to the presence of multiple bands, QIAquick Gel Extraction kit (QiaGen) was used following manufacturer’s instructions. When the quality of the DNA was high (a unique amplicon of above 20 ng/μL concentration), purifications were done using Exosap, Illustra ExoStar-1-Step (GE Healthcare, Buckinghamshire, UK) following the instructions of the manufacturers. Purified amplicons with a concentration of 20 ng/μL or more were sent to Macrogen (Korea) for Sanger sequencing with primers used in the amplification.

### Phylogenetic analyses

Consensus sequences were obtained using Geneious version 9.0.2 http://www.geneious.com^38^. Subsequently, they were subjected to a BLAST search with megablast option and compared against the sequences in the National Center for Biotechnology Information (NCBI) nucleotide databases^39^ to check for contamination. Evaluation of EMBL/GenBank/DDBJ databases for ITS nrDNA and nrLSU sequences of a large set of *Xylodon* species was performed to provide a phylogenetic framework to *X*. *australis* and to maximize the molecular information available for these taxa (See Suplementary Table S1 on line). Three specimens of the sister genus *Lyomyces* P. Karst. were included as outgroup in the phylogenetic analyses^40^ The maximum parsimony (MP), maximum likelihood (ML), and Bayesian inference analyses of specimens in the S1 Table are also deposited in protocols.io under the doi mentioned above. The ML and Bayesian analyses were done with the general time reversible model^41^, including estimation of invariant sites and a discrete gamma distribution with six categories (GTR + G), as selected by PAUP*Version 4.0b10.

Individual datasets of ITS nrDNA, nrLSU and a combined alignment of ITS nrDNA + nrLSU regions were used to compare specimens of *Xylodon australis* from South America and Australia with other *Xylodon* species. In the combined alignment, only samples with complete ITS nrDNA and nrLSU sequences were included to improve the resolution power of the obtained phylogeny.

### Statistical tests for morphological traits

Basidia and spore morphology were analyzed in order to assess the morphological difference found in previous studies for *Xylodon australis* specimens from different locations^3^. One-way ANOVA tests were performed between *X*. *australis* lineages determined by molecular phylogenetic analyses.

### Inferring the position of the nomeclatural type of *Xylodon australis* in the molecular phylogenetic tree

In order to determinate the position of the type specimen of *Xylodon australis* in the molecular phylogenetic tree using phenotypic traits, we used the methodology proposed by Revell et al.^12^. First, ultrametric phylogenetic trees of *X*. *australis* and closely related species were estimated using BI implemented in BEAST v2.4.3^42,43^ for each alignment. Site model partition and GTR + G substitution model was selected individually for both, ITS nrDNA and nrLSU regions in all datasets using BEAUti v2.4.3 interface^43^. Birth-Dead model was used as tree prior. Three independent MCMC runs were specified for 50 million generations, sampling every 5000th generation. Tree and log files were combined in Logcombiner v.1.7 and results were visualized in Tracer v.1.6^44^, to evaluate whether the effective sample size (ESS) values were above 200 and to check for parameter convergence. The resulting trees were summarized in a maximum clade credibility tree by TreeAnnotator v.1.7.^43^ with a burnin of 5000 trees for each run.

Second, the subtree formed by the crown group for all *Xylodon australis* specimens analyzed was selected as a base tree for the next analyses. Five continuous morphological traits usually known as taxonomically informative for Hymenochaetales were selected: basidia length and width, spore length and width, and spore length–width ratio^45^. These traits were measured for the *X*. *australis* type specimen and for all samples in the subtree. To place the type specimen of *X*. *australis* into the molecular tree using the continuous morphological traits, the function *locate.yeti* from the R package *phytools* (v0.6.60) was used^16^. This function adapts the approach proposed by Felsenstein^14,16^ to estimate phylogeny from continuous traits using a maximum likelihood framework. To include more than one continuous trait, a phylogenetic principal component analysis is performed first^46^. Then, these principal components are used to identify the optimal position of the type specimen in the phylogenetic tree applying the maximum likelihood criterion (see Equation 1 in Revell et al.^12^). The method relies on the assumption that the characters have evolved along the tree and that the morphological differences between species are mostly due to inherited genetic differences.

In order to assess the performance of this approach, a randomization test with the original pure molecular subtree was conducted. We ran 100 replicates in which we pruned one tree-tip at random per replicate. Then, the tip was included again in the tree in two ways: randomly located or using the *locate.yeti* function. These two trees were compared with the original molecular tree by computing branch-score^47^ and quadratic path distances^48^ using the R package *phangorn*^49^. A lower distance means more similarity of the reconstructed tree with the actual molecular tree. In addition, to test the hypothesis that the inferred ML phylogenetic position of the type specimen through *locate.yeti* function is significantly better than alternative locations, a likelihood ratio test was conducted by comparing the likelihood score of tree in which type position was constrained, to trees with unconstrained type locations. Simulations of continuous traits were performed on the constrained tree, and then type position was inferred without constraint. These simulations were used to generate a null distribution to check for significance of the likelihood ratio test^12^.

The same methodology was applied to each specimen from New Zealand (PDD Herbarium) for which no molecular data were obtained.

## Supplementary Information

Supplementary Information.

Supplementary References.
