# Supplementary material for: Linking morphological and molecular sources to disentangle the case of Xylodon australis
Source: Sci Rep. 2020 Dec 15;10:22004. doi: 10.1038/s41598-020-78399-8 (PMC7738490; doi:10.1038/s41598-020-78399-8)
Supplement: Supplementary file 1 — Supplementary Information. [file 41598_2020_78399_MOESM1_ESM.pdf]

Linking morphological and molecular sources to disentangle the case of *Xylodon australis*

Javier Fernández-López[1,3], M. Teresa Telleria[1], Margarita Dueñas[1], Mara Laguna-Castro[1,4], Klaus Schliep[2], María P. Martín[1]

[1] Department of Mycology, Real Jardín Botánico-CSIC, Plaza de Murillo 2. 28014 Madrid, Spain
corresponding author e-mail: jflopez@rjb.csic.es

[2] Graz University of Technology, Austria

[3] Instituto de Investigación en Recursos Cinegéticos, IREC (UCLM-CSIC-JCCM), Ciudad Real, Spain

[4] Centro de Astrobiología (INTA-CSIC), Instituto Nacional de Técnica Aeroespacial Esteban Terradas, Torrejón de Ardoz, Spain

Table S1: Specimens and sequences included to the phylogenetic analyses. Data of voucher, and publication if available. New sequences obtained in this study in bold. (-) Sequences not obtained or nor available in GenBank.

Species	Specimen voucher	GenBank Acc. Nº.		Reference
		ITS	LSU	
Xylodon apacherensis (Gilb. & Canf.) Hjorstam & Ryvarden	Canfield 180, holotype	KY081800	-	[29]
X. asperus (Fr.) Hjorstam & Ryvarden	KH Nilsson s.n.	DQ873606	DQ873607	[50]
	Larsson 8530 (GB)	-	AY586675	[51]
	UC2023169	KP814365	-	[29]
X. astrocystidiatus (Yurchenko & Sheng H. Wu) Riebesehl, Yurchenko & E. Langer	Wu 9211-71	JN129972	JN129973	[52]
X. attenuatus Spirin & Viner	Spirin 8775, holotype	MH324476	-	[53]
X. australis (Berk.) Hjorstam & Ryvarden	CANB569566	MT158734	-	**This study**
	CANB569567	MT158703	MT158739	**This study**
	CANB569569	MT158704	MT158740	**This study**
	CANB569570	MT158735	-	**This study**
	CANB569571	MT158705	MT158741	**This study**
	CANB627912	MT158706	MT158742	**This study**
	CANB642810	MT158707	MT158743	**This study**
	CANB649539	MT158736	-	**This study**
	CANB751963	MT158708	MT158744	**This study**
	CANB752080	MT158709	MT158745	**This study**
	CANB752088	MT158710	MT158746	**This study**
	CANB752161	MT158711	MT158747	**This study**
	CANB791229	MT158712	MT158748	**This study**
	CANB791360	MT158713	MT158749	**This study**
	CANB791385	MT158714	MT158750	**This study**
	CANB791543	MT158737	-	**This study**
	CANB869100	MT158715	MT158751	**This study**
	CANB869124	MT158716	MT158752	**This study**
X. borealis (Kotir. & Saaren.) Hjorstam & Ryvarden	JS26064	-	AY586677	[51]
	Spirin 9416	MH317760	MH638259	[53]
	UC2022850	KP814307	-	[54]
X. brevisetus (P. Karst.) Hjorstam & Ryvarden	JS17863	-	AY586676	[51]
	Larsson 12386 (GB)	DQ873612	DQ873612	[17]
	UC2023199	KP814485	-	Unpublished
X. bubalinus (Ming Wang, Yuan Y. Chen & B.K. Cui) C.C. Chen & Sheng H. Wu	CL Zhao 184	MG231628	-	Unpublished
	Cui 6834	KY290981	-	[55]
	Cui 12887	KY290982	-	[55]
	Cui 1288, holotype	KY290983	-	[55]
X. chinensis (C.C. Chen & Sheng H. Wu) C.C.Chen & Sheng H. Wu	Wu 1307-42	KX857802	-	[56]
	Wu 1407.105, holotype	KX857804	KX857811	[56]

X. crystalliger Viner	KUN2312, holotype	MH324477	-	[52]
X. cystidiatus (A. David & Rajchenb.) Riebesehl & Langer	FR-0249200	MH880195	MH884896	[17]
X. detriticus (Bourdot) K.H. Larss.,Viner & Spirin	UC203108	KP814412	-	[54]
	Zíbarová 30.10.17	MH320793	MH651372	[53]
X. dimiticus (Jia J. Chen & LW. Zhou) Riebesehl & Langer	Dai 15321	KT989969	-	[57]
X. exilis Yurchenko, Riebesehl & E. Larger	MSK-F 7381	MH880196	MH884897	[17]
	MSK-F 7431	-	-	[17]
	TUB-FO 42450	MH880197	-	[17]
	TUB-FO 42565, holotype	MH880198	MH884898	[17]
X. filicinus Yurchenko & Riebesehl	MSK-F12869, holotype	MH880199	MH884899	[17]
	MSK-F 12870	MH880200	MH884900	[17]
X. flaviporus (Berk. & M.A. Curtis ex Cooke) Riebesehl & E. Langer	FR-0249797	MH880201	MH884901	[17]
	FUG 1053	AF145575	-	[58]
	ICMP 13836	AF145585	-	[58]
	GEL3462 (KAS)	MH880202	-	[17]
	GEL5047 (KAS)	MH880203	-	[17]
	KUC20130808-17	-	KJ668314	[59]
X. follis Riebesehl, Yurchenko &E. Langer	FR-0249814, holotype	MH880204	MH884902	[17]
X. hastifer (Hjorstam & Ryvarden) Hjorstam & Ryvarden	Ryvarden 19767, holotype	KY081801	-	[29]
X. heterocystidiatus (H.X. Xiong, Y.C. Dai & Seng H. Wu) Riebesehl, Yurchenko & E. langer	Wu 9209-27	JX175045	KX857821	[52, 56]
X. hyphodontinus (Hjorstam & Ryvarden) Riebesehl,Yurchenko & G. Gruhn	GEL9222 (KAS)	MH880205	MH884903	[17]
	LIP GG-GUY13-044	MH880206	MH884904	[17]
	LIP GG-MAR12-238	MH880207	MH884905	[17]
	LIP GG-MAR15-127	MH880208	MH884906	[17]
X. lenis Hjorstam & Ryvarden	Wu 0808-32	JX175043	KX857820	[52]
	Wu 890714-3, isotype	KY081802	-	[29]
	Cui 5950	KT989972	-	Unpublished
X. magallanesii sp. nov.	14120MD (MA-Fungi 90391)	MT158720	MT158756	**This study**
	14164MD (MA-Fungi 90393)	MT158721	MT158757	**This study**
	14629MD (MA-Fungi 91815)	MT158722	MT158758	**This study**
	15630MD (MA-Fungi 91816)	MT158723	MT158759	**This study**
	15632MD (MA-Fungi 91817)	MT158724	MT158760	**This study**
	15634MD (MA-Fungi 91818)	MT158725	MT158761	**This study**
	15637MD (MA-Fungi 91819)	MT158726	MT158762	**This study**
	15638MD (MA-Fungi 91820)	MT158727	MT158763	**This study**
	20007Tell. (MA-Fungi 91821)	MT158728	MT158764	**This study**
	20008Tell. (MA-Fungi 90397), holotype	MT158729	MT158765	**This study**
	AG 730	MT158730	MT158766	**This study**
	AG 1548	MT158731	MT158767	**This study**
	AG 1872	MT158732	MT158768	**This study**
	CIEFAP-11041 (CFMR)	-	MH884895	[17]

	MES-2880	MH930235	-	Unpublished
	MR 11041 (PDD 69093)	MT158733	MT158769	**This study**
X. mollissimus (L.W. Zhou) C.C. Chen & Sheng H. Wu	LWZ20160318-3, holotype	KY007517	-	[60]
X. nespori (Bres.) Hjorstam & Ryvarden	GEL3158 (KAS)	DQ340310	DQ340346	Unpublished
	GEL3290 (KAS)	DQ340309	DQ340343	Unpublished
	GEL3302 (KAS)	DQ340308	DQ340344	Unpublished
	GEL3309 (KAS)	DQ340307	DQ340345	[52]
	JR14 (KAS)	MH880210	-	[17]
	B. Nordon 030915 (GB)	DQ873622	-	[50]
	KUC20161012-5	MF774797	-	Unpublished
	19275Tell. (MA-Fungi 79920)	MT158717	MT158753	**This study**
X. niemelaei (Sheng H. Wu) Hjorstam & Ryvarden	Dai 15358	KT989973	-	[57]
	FR-0219860	MH880211	-	[17]
	FR-0249174	MH880212	-	[17]
	FR-024178	-	MH884907	[17]
	GC 1508-146	KX857798	-	[56]
	GEL4998 (KAS)	EU583422	DQ340348	Unpublished
	Wu 1010-62	-	KX857817	[56]
X. nongravis (Lloyd) C.C. Chen & Sheng H. Wu	CHWC1506-2	KX857800	-	[56]
	GC1412-22	KX857801	KX857818	[56]
	Spirin 5763	MH324469	MH656724	[53]
X. nothofagi (G. Cunn.) Hjorstam & Ryvarden	PDD 91630	GQ411524	-	[61]
X. ovisporus (Corner) Riebesehl & E. Langer	GEL3493 (KAS)	EU583421	-	Unpublished
	ICMP 13835	-	MH260063	[40]
	KUC20130725-29	KJ668513	KJ668365	[59]
X. paradoxus (Schrad.) Chevall.	FCUG 1517	AF145572	-	[58]
	FCUG 2425	AF145571	-	[58]
	GEL2511 (KAS)	-	AF518647	[62]
	JR06 (KAS)	MH880219	-	[17]
	JR28 (KAS)	-	MH884908	[17]
	Miettinen 7978	FN907912	FN907912	[63]
X. pruinosus (Bres.) Spirin & Viner	Spirin 2877	MH332700	-	[53]
	UC2023108	KP814412	-	[54]
X. pseudolanatus Nakasone, Yurchenko & Riebesehl	FP-150922 (CFMR), holotype	MH880220	MH884909	[17]
X. pseudotropicus (C.L. Zhao, B.K. Cui & Y.C. Dai) Riebesehl, Yurchenko & E. Langer	Dai 10768, holotype	KF917543	-	[64]
X. quercinus (Pers.) Gray	5556MD (MA-Fungi 27435)	MT158718	MT158754	**This study**
	Kotiranta 27060	MH320792	-	[53]
	Larsson 11076	KT361633	AY586678	[27, 51]
	Miettinen 15050.1 (H 6013352)	KT361632	-	[65]
	SPG 0826 (MA-Fungi 84446)	MT158719	MT158755	**This study**
X. raduloides Riebesehl & E. Langer	Dai 12631	KT203307	KT203328	unpublished
	FCUG 2239	-	AF141613	[66]
	ICMP 13833	AF145580	-	[58]
	JR 26 (KAS)	MH880225	MH884910	[17]

	LR 18813	MH880226	MH884911	[17]
X. ramicida Spirin & Miettinen	Spirin 7664, holotype	KT361634	-	[65]
X. reticulatus (C.C. Chen & Sheng H. Wu) C.C. Chen & Sheng H. Wu	GC 1512-1	KX857808	KX857813	[56]
	KUC20160721B-26	MF774798	-	[67]
	Wu 1109-178, holotype	KX857805	-	[56]
X. rhizomorphus (C.L. Zhao, B.K. Cui & Y.C. Dai) Riebesehl, Yurchenko & E. Langer	Dai 12354	KF917544	-	[64]
	Dai 12367, holotype	KF917545	-	[64]
	Dai 12389	KF917546	-	[64]
X. rimosissimus (Peck) Hjorstam & Ryvarden	DLL2011-081 (CFMR)	KJ140600	-	[68]
	Ryberg 021031 (GB)	DQ873627	DQ873628	[50]
X. serpentiformis (Langer) Hjorstam & Ryvarden	GEL2668 (KAS)	MH880227	-	[17]
	FO 40675 (TUB)	MH880228	-	[17]
	FO 40985 (TUB)	-	MH884912	[17]
	FO 42688 (TUB)	MH880229	MH884913	[17]
X. spathulatus (Schrad.) Kuntze	GEL2960 (KAS)	KY081803	-	[29]
	Larsson 7085 (GB)	KY081804	-	[29]
	F 12931 (MSK)	MH880231	MH884914	[17]
	MMS7224 (KAS)	MH880230	-	[17]
X. subclavatus (Yurchenko, H.X. Xiong & Sheng H. Wu) Riebesehl, Yurchenko & E. Langer	FO 42167 (TUB)	MH880232	-	[17]
X. subflaviporus C.C. Chen & Sheng H. Wu	GEL 3466 (KAS)	MH880253	-	[17]
	Wu 0809-76	KX857803	KX857815	[56]
X. subtropicus (C.C. Chen & Sheng H. Wu) C.C. Chen & Sheng H. Wu	Wu 1508-2	KX857806	KX857812	[56]
	Wu 9806-105, holotype	KX857807	KX857809	[56]
X. ussuriensis Viner	KUN1989, holotype	MH324468	-	[53]
X. verecundus (G. Cunn.) Yurchencho & Riebesehl	Larsson 1221 (GB)	DQ873642	-	[50]
**Outgroup**				
Lyomyces crustosus (Pers.) P. Karst.	GEL2325 (KAS)	DQ340313	DQ340354	Unpublished
	GEL5360 (KAS)	DQ340315	DQ340355	Unpublished
	GEL5336 (KAS)	DQ340314	DQ340356	Unpublished

Figure S1

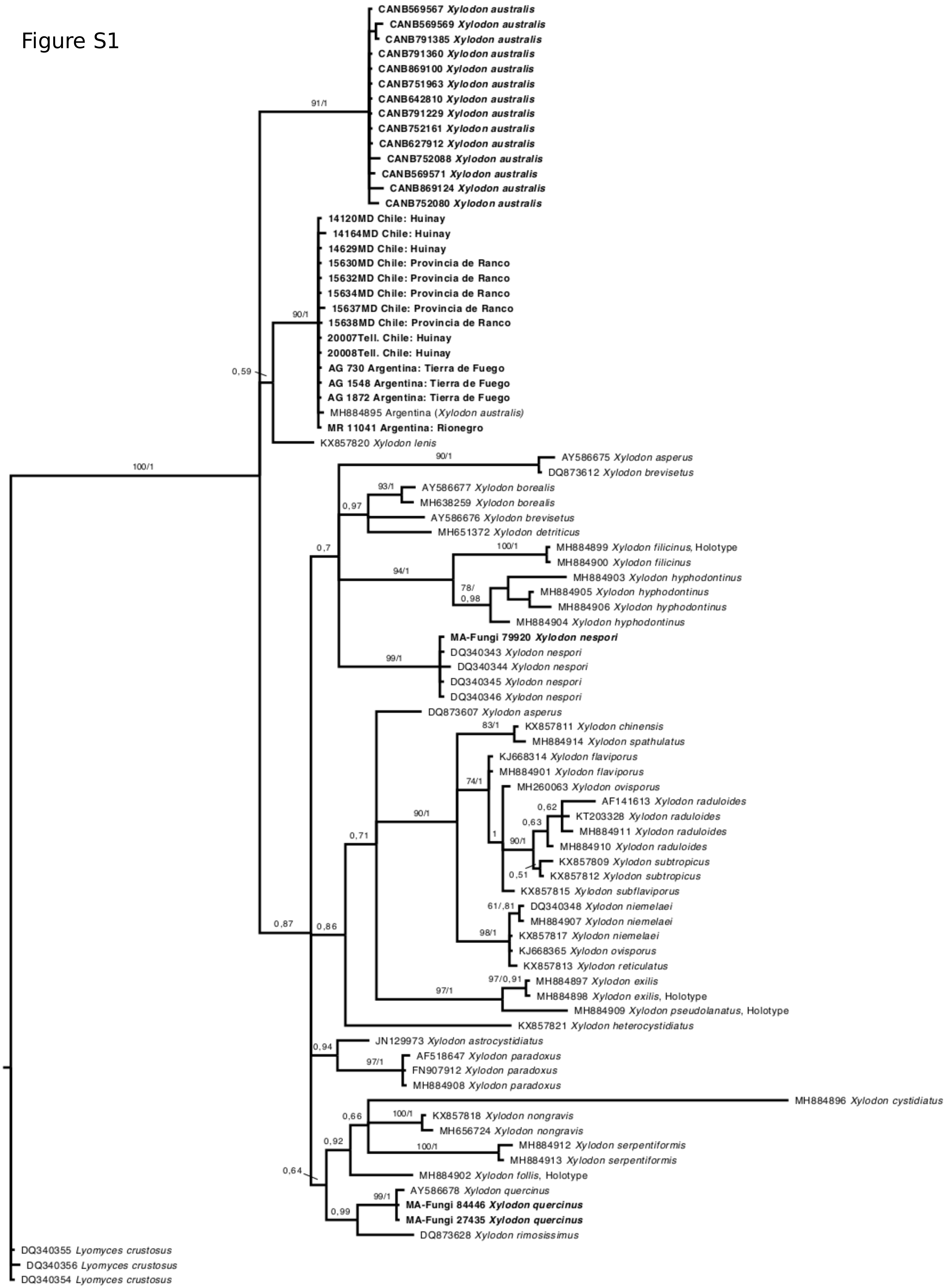

CANB569567 Xylodon australis
CANB569569 Xylodon australis
CANB791385 Xylodon australis
CANB791360 Xylodon australis
CANB869100 Xylodon australis
CANB751963 Xylodon australis
CANB642810 Xylodon australis
CANB791229 Xylodon australis
CANB752161 Xylodon australis
CANB627912 Xylodon australis
CANB752088 Xylodon australis
CANB569571 Xylodon australis
CANB869124 Xylodon australis
CANB752080 Xylodon australis
91/1
14120MD Chile: Huinay
14164MD Chile: Huinay
14629MD Chile: Huinay
15630MD Chile: Provincia de Ranco
15632MD Chile: Provincia de Ranco
15634MD Chile: Provincia de Ranco
15637MD Chile: Provincia de Ranco
15638MD Chile: Provincia de Ranco
20007Tell. Chile: Huinay
20008Tell. Chile: Huinay
AG 730 Argentina: Tierra de Fuego
AG 1548 Argentina: Tierra de Fuego
AG 1872 Argentina: Tierra de Fuego
MH884895 Argentina (Xylodon australis)
MR 11041 Argentina: Rionegro
90/1
0,59
KX857820 Xylodon lenis
100/1
AY586675 Xylodon asperus
DQ873612 Xylodon brevisetus
90/1
AY586677 Xylodon borealis
MH638259 Xylodon borealis
93/1
AY586676 Xylodon brevisetus
MH651372 Xylodon detriticus
0,97
0,7
MH884899 Xylodon filicinus, Holotype
MH884900 Xylodon filicinus
100/1
MH884903 Xylodon hyphodontinus
MH884905 Xylodon hyphodontinus
MH884906 Xylodon hyphodontinus
MH884904 Xylodon hyphodontinus
94/1
78/ 0,98
MA-Fungi 79920 Xylodon nespori
DQ340343 Xylodon nespori
DQ340344 Xylodon nespori
DQ340345 Xylodon nespori
DQ340346 Xylodon nespori
99/1
DQ873607 Xylodon asperus
KX857811 Xylodon chinensis
MH884914 Xylodon spathulatus
83/1
KJ668314 Xylodon flaviporus
MH884901 Xylodon flaviporus
MH260063 Xylodon ovisporus
AF141613 Xylodon raduloides
KT203328 Xylodon raduloides
MH884911 Xylodon raduloides
MH884910 Xylodon raduloides
KX857809 Xylodon subtropicus
KX857812 Xylodon subtropicus
KX857815 Xylodon subflaviporus
0,62
0,63
90/1
0,51
1
74/1
90/1
0,71
DQ340348 Xylodon niemelaei
MH884907 Xylodon niemelaei
KX857817 Xylodon niemelaei
KJ668365 Xylodon ovisporus
KX857813 Xylodon reticulatus
61/,81
98/1
MH884897 Xylodon exilis
MH884898 Xylodon exilis, Holotype
97/0,91
MH884909 Xylodon pseudolanatus, Holotype
97/1
KX857821 Xylodon heterocystidiatus
0,87
0,86
JN129973 Xylodon astrocystidiatus
AF518647 Xylodon paradoxus
FN907912 Xylodon paradoxus
MH884908 Xylodon paradoxus
97/1
0,94
MH884896 Xylodon cystidiatus
KX857818 Xylodon nongravis
MH656724 Xylodon nongravis
100/1
0,66
MH884912 Xylodon serpentiformis
MH884913 Xylodon serpentiformis
100/1
0,92
MH884902 Xylodon follis, Holotype
0,64
AY586678 Xylodon quercinus
MA-Fungi 84446 Xylodon quercinus
MA-Fungi 27435 Xylodon quercinus
99/1
0,99
DQ873628 Xylodon rimosissimus
DQ340355 Lyomyces crustosus
DQ340356 Lyomyces crustosus
DQ340354 Lyomyces crustosus

Figure S2

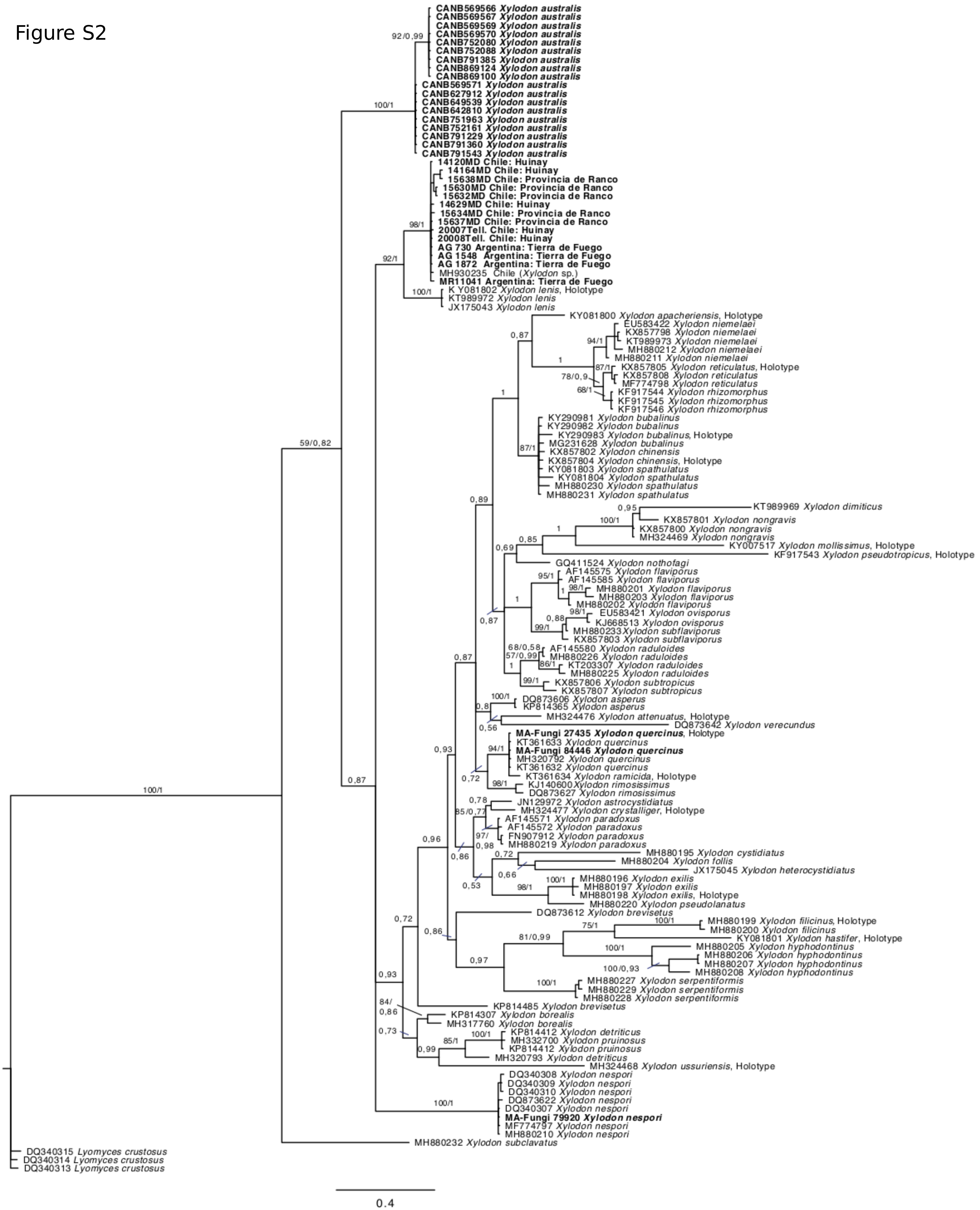

Figure S3

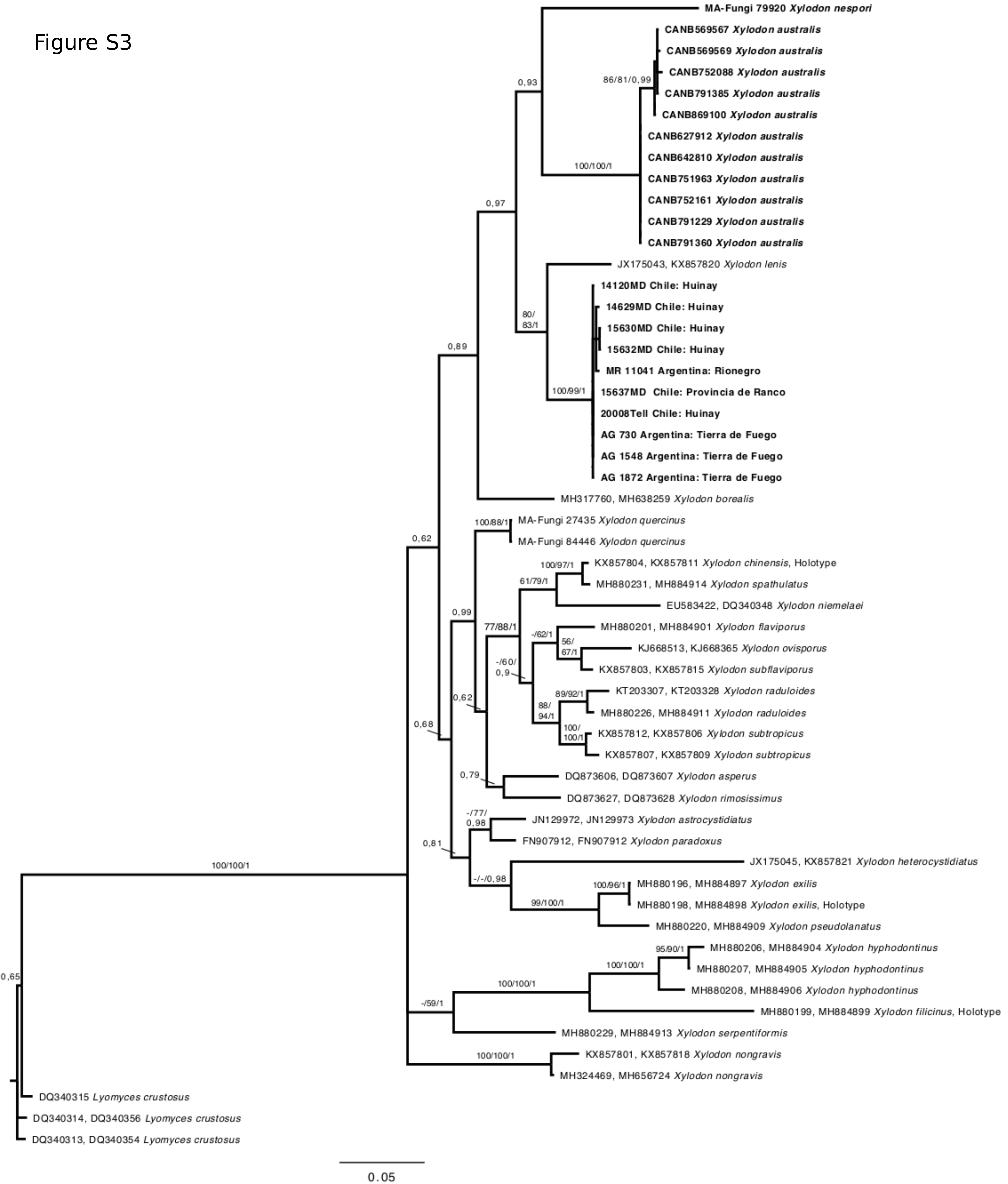

MA-Fungi 79920 Xylodon nespori
CANB569567 Xylodon australis
CANB569569 Xylodon australis
CANB752088 Xylodon australis
CANB791385 Xylodon australis
CANB869100 Xylodon australis
CANB627912 Xylodon australis
CANB642810 Xylodon australis
CANB751963 Xylodon australis
CANB752161 Xylodon australis
CANB791229 Xylodon australis
CANB791360 Xylodon australis
JX175043, KX857820 Xylodon lenis
14120MD Chile: Huinay
14629MD Chile: Huinay
15630MD Chile: Huinay
15632MD Chile: Huinay
MR 11041 Argentina: Rionegro
15637MD Chile: Provincia de Ranco
20008Tell Chile: Huinay
AG 730 Argentina: Tierra de Fuego
AG 1548 Argentina: Tierra de Fuego
AG 1872 Argentina: Tierra de Fuego
MH317760, MH638259 Xylodon borealis
MA-Fungi 27435 Xylodon quercinus
MA-Fungi 84446 Xylodon quercinus
KX857804, KX857811 Xylodon chinensis, Holotype
MH880231, MH884914 Xylodon spathulatus
EU583422, DQ340348 Xylodon niemelaei
MH880201, MH884901 Xylodon flaviporus
KJ668513, KJ668365 Xylodon ovisporus
KX857803, KX857815 Xylodon subflaviporus
KT203307, KT203328 Xylodon raduloides
MH880226, MH884911 Xylodon raduloides
KX857812, KX857806 Xylodon subtropicus
KX857807, KX857809 Xylodon subtropicus
DQ873606, DQ873607 Xylodon asperus
DQ873627, DQ873628 Xylodon rimosissimus
JN129972, JN129973 Xylodon astrocystidiatus
FN907912, FN907912 Xylodon paradoxus
JX175045, KX857821 Xylodon heterocystidiatus
MH880196, MH884897 Xylodon exilis
MH880198, MH884898 Xylodon exilis, Holotype
MH880220, MH884909 Xylodon pseudolanatus
MH880206, MH884904 Xylodon hyphodontinus
MH880207, MH884905 Xylodon hyphodontinus
MH880208, MH884906 Xylodon hyphodontinus
MH880199, MH884899 Xylodon filicinus, Holotype
MH880229, MH884913 Xylodon serpentiformis
KX857801, KX857818 Xylodon nongravis
MH324469, MH656724 Xylodon nongravis
DQ340315 Lyomyces crustosus
DQ340314, DQ340356 Lyomyces crustosus
DQ340313, DQ340354 Lyomyces crustosus
0,93
86/81/0,99
100/100/1
0,97
80/
83/1
100/99/1
0,89
0,62
100/88/1
100/97/1
61/79/1
0,99
77/88/1
-/62/1
56/
67/1
-/60/
0,9
89/92/1
88/
94/1
100/
100/1
0,62
0,68
0,79
-/77/
0,98
0,81
-/-/0,98
100/96/1
99/100/1
100/100/1
95/90/1
100/100/1
100/100/1
0,65
-/59/1
100/100/1
0.05

Figure S4

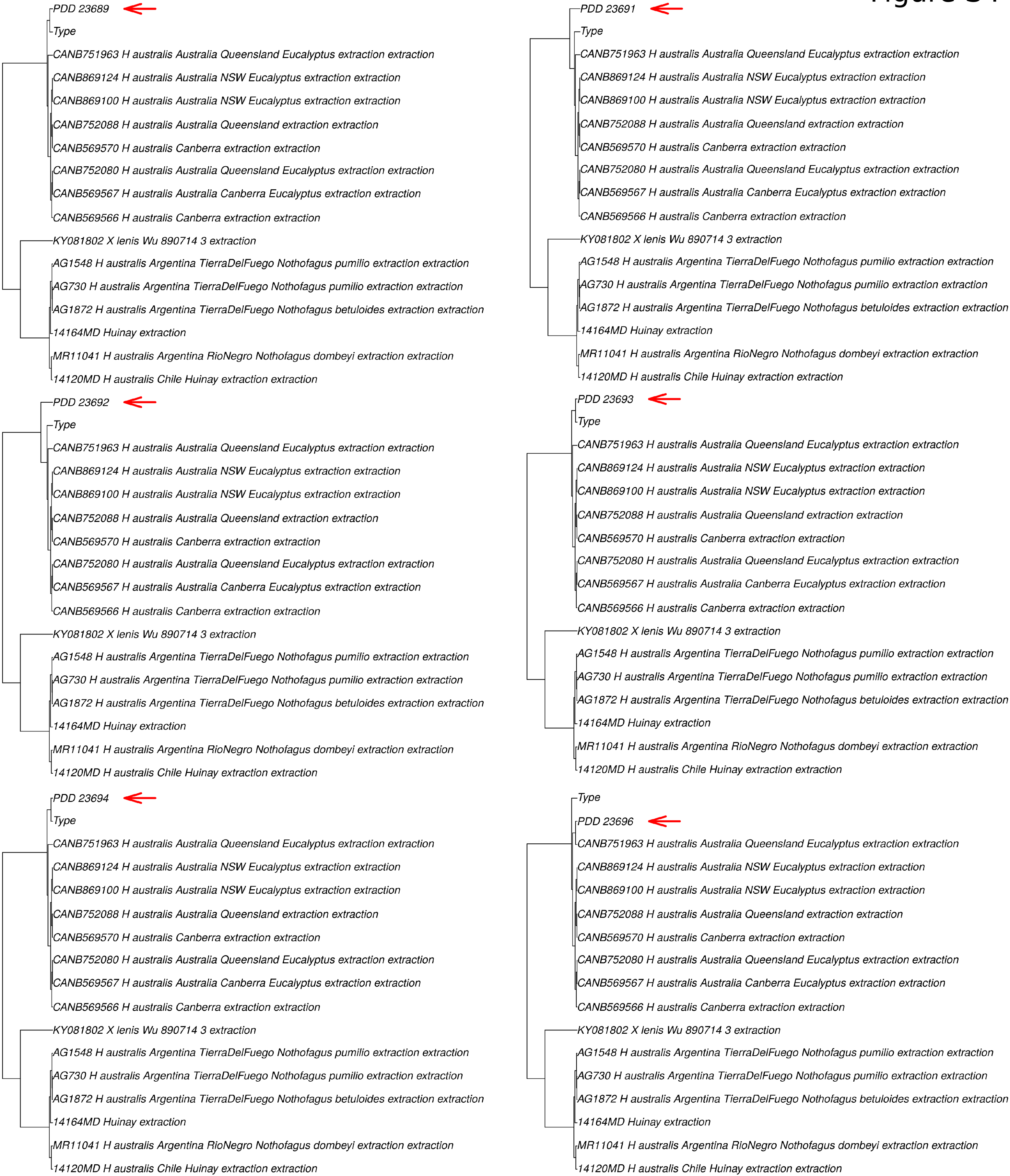
PDD 23689
Type
CANB751963 H australis Australia Queensland Eucalyptus extraction extraction
CANB869124 H australis Australia NSW Eucalyptus extraction extraction
CANB869100 H australis Australia NSW Eucalyptus extraction extraction
CANB752088 H australis Australia Queensland extraction extraction
CANB569570 H australis Canberra extraction extraction
CANB752080 H australis Australia Queensland Eucalyptus extraction extraction
CANB569567 H australis Australia Canberra Eucalyptus extraction extraction
CANB569566 H australis Canberra extraction extraction
KY081802 X lenis Wu 890714 3 extraction
AG1548 H australis Argentina TierraDelFuego Nothofagus pumilio extraction extraction
AG730 H australis Argentina TierraDelFuego Nothofagus pumilio extraction extraction
AG1872 H australis Argentina TierraDelFuego Nothofagus betuloides extraction extraction
14164MD Huinay extraction
MR11041 H australis Argentina RioNegro Nothofagus dombeyi extraction extraction
14120MD H australis Chile Huinay extraction extraction
PDD 23691
Type
CANB751963 H australis Australia Queensland Eucalyptus extraction extraction
CANB869124 H australis Australia NSW Eucalyptus extraction extraction
CANB869100 H australis Australia NSW Eucalyptus extraction extraction
CANB752088 H australis Australia Queensland extraction extraction
CANB569570 H australis Canberra extraction extraction
CANB752080 H australis Australia Queensland Eucalyptus extraction extraction
CANB569567 H australis Australia Canberra Eucalyptus extraction extraction
CANB569566 H australis Canberra extraction extraction
KY081802 X lenis Wu 890714 3 extraction
AG1548 H australis Argentina TierraDelFuego Nothofagus pumilio extraction extraction
AG730 H australis Argentina TierraDelFuego Nothofagus pumilio extraction extraction
AG1872 H australis Argentina TierraDelFuego Nothofagus betuloides extraction extraction
14164MD Huinay extraction
MR11041 H australis Argentina RioNegro Nothofagus dombeyi extraction extraction
14120MD H australis Chile Huinay extraction extraction
PDD 23692
Type
CANB751963 H australis Australia Queensland Eucalyptus extraction extraction
CANB869124 H australis Australia NSW Eucalyptus extraction extraction
CANB869100 H australis Australia NSW Eucalyptus extraction extraction
CANB752088 H australis Australia Queensland extraction extraction
CANB569570 H australis Canberra extraction extraction
CANB752080 H australis Australia Queensland Eucalyptus extraction extraction
CANB569567 H australis Australia Canberra Eucalyptus extraction extraction
CANB569566 H australis Canberra extraction extraction
KY081802 X lenis Wu 890714 3 extraction
AG1548 H australis Argentina TierraDelFuego Nothofagus pumilio extraction extraction
AG730 H australis Argentina TierraDelFuego Nothofagus pumilio extraction extraction
AG1872 H australis Argentina TierraDelFuego Nothofagus betuloides extraction extraction
14164MD Huinay extraction
MR11041 H australis Argentina RioNegro Nothofagus dombeyi extraction extraction
14120MD H australis Chile Huinay extraction extraction
PDD 23693
Type
CANB751963 H australis Australia Queensland Eucalyptus extraction extraction
CANB869124 H australis Australia NSW Eucalyptus extraction extraction
CANB869100 H australis Australia NSW Eucalyptus extraction extraction
CANB752088 H australis Australia Queensland extraction extraction
CANB569570 H australis Canberra extraction extraction
CANB752080 H australis Australia Queensland Eucalyptus extraction extraction
CANB569567 H australis Australia Canberra Eucalyptus extraction extraction
CANB569566 H australis Canberra extraction extraction
KY081802 X lenis Wu 890714 3 extraction
AG1548 H australis Argentina TierraDelFuego Nothofagus pumilio extraction extraction
AG730 H australis Argentina TierraDelFuego Nothofagus pumilio extraction extraction
AG1872 H australis Argentina TierraDelFuego Nothofagus betuloides extraction extraction
14164MD Huinay extraction
MR11041 H australis Argentina RioNegro Nothofagus dombeyi extraction extraction
14120MD H australis Chile Huinay extraction extraction
PDD 23694
Type
CANB751963 H australis Australia Queensland Eucalyptus extraction extraction
CANB869124 H australis Australia NSW Eucalyptus extraction extraction
CANB869100 H australis Australia NSW Eucalyptus extraction extraction
CANB752088 H australis Australia Queensland extraction extraction
CANB569570 H australis Canberra extraction extraction
CANB752080 H australis Australia Queensland Eucalyptus extraction extraction
CANB569567 H australis Australia Canberra Eucalyptus extraction extraction
CANB569566 H australis Canberra extraction extraction
KY081802 X lenis Wu 890714 3 extraction
AG1548 H australis Argentina TierraDelFuego Nothofagus pumilio extraction extraction
AG730 H australis Argentina TierraDelFuego Nothofagus pumilio extraction extraction
AG1872 H australis Argentina TierraDelFuego Nothofagus betuloides extraction extraction
14164MD Huinay extraction
MR11041 H australis Argentina RioNegro Nothofagus dombeyi extraction extraction
14120MD H australis Chile Huinay extraction extraction
Type
PDD 23696
CANB751963 H australis Australia Queensland Eucalyptus extraction extraction
CANB869124 H australis Australia NSW Eucalyptus extraction extraction
CANB869100 H australis Australia NSW Eucalyptus extraction extraction
CANB752088 H australis Australia Queensland extraction extraction
CANB569570 H australis Canberra extraction extraction
CANB752080 H australis Australia Queensland Eucalyptus extraction extraction
CANB569567 H australis Australia Canberra Eucalyptus extraction extraction
CANB569566 H australis Canberra extraction extraction
KY081802 X lenis Wu 890714 3 extraction
AG1548 H australis Argentina TierraDelFuego Nothofagus pumilio extraction extraction
AG730 H australis Argentina TierraDelFuego Nothofagus pumilio extraction extraction
AG1872 H australis Argentina TierraDelFuego Nothofagus betuloides extraction extraction
14164MD Huinay extraction
MR11041 H australis Argentina RioNegro Nothofagus dombeyi extraction extraction
14120MD H australis Chile Huinay extraction extraction

Figure S4

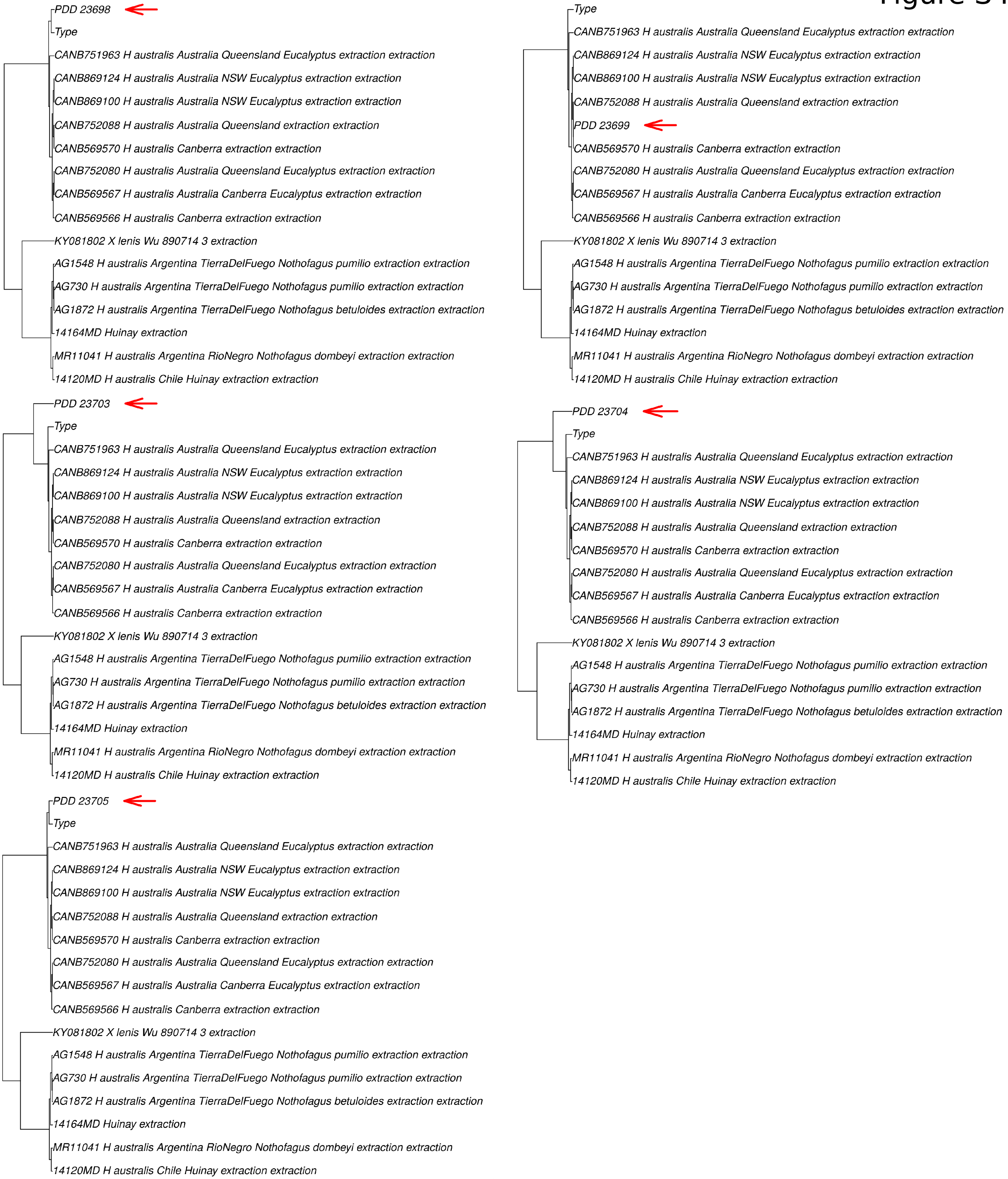
PDD 23698
Type
CANB751963 H australis Australia Queensland Eucalyptus extraction extraction
CANB869124 H australis Australia NSW Eucalyptus extraction extraction
CANB869100 H australis Australia NSW Eucalyptus extraction extraction
CANB752088 H australis Australia Queensland extraction extraction
CANB569570 H australis Canberra extraction extraction
CANB752080 H australis Australia Queensland Eucalyptus extraction extraction
CANB569567 H australis Australia Canberra Eucalyptus extraction extraction
CANB569566 H australis Canberra extraction extraction
KY081802 X lenis Wu 890714 3 extraction
AG1548 H australis Argentina TierraDelFuego Nothofagus pumilio extraction extraction
AG730 H australis Argentina TierraDelFuego Nothofagus pumilio extraction extraction
AG1872 H australis Argentina TierraDelFuego Nothofagus betuloides extraction extraction
14164MD Huinay extraction
MR11041 H australis Argentina RioNegro Nothofagus dombeyi extraction extraction
14120MD H australis Chile Huinay extraction extraction
Type
CANB751963 H australis Australia Queensland Eucalyptus extraction extraction
CANB869124 H australis Australia NSW Eucalyptus extraction extraction
CANB869100 H australis Australia NSW Eucalyptus extraction extraction
CANB752088 H australis Australia Queensland extraction extraction
PDD 23699
CANB569570 H australis Canberra extraction extraction
CANB752080 H australis Australia Queensland Eucalyptus extraction extraction
CANB569567 H australis Australia Canberra Eucalyptus extraction extraction
CANB569566 H australis Canberra extraction extraction
KY081802 X lenis Wu 890714 3 extraction
AG1548 H australis Argentina TierraDelFuego Nothofagus pumilio extraction extraction
AG730 H australis Argentina TierraDelFuego Nothofagus pumilio extraction extraction
AG1872 H australis Argentina TierraDelFuego Nothofagus betuloides extraction extraction
14164MD Huinay extraction
MR11041 H australis Argentina RioNegro Nothofagus dombeyi extraction extraction
14120MD H australis Chile Huinay extraction extraction
PDD 23703
Type
CANB751963 H australis Australia Queensland Eucalyptus extraction extraction
CANB869124 H australis Australia NSW Eucalyptus extraction extraction
CANB869100 H australis Australia NSW Eucalyptus extraction extraction
CANB752088 H australis Australia Queensland extraction extraction
CANB569570 H australis Canberra extraction extraction
CANB752080 H australis Australia Queensland Eucalyptus extraction extraction
CANB569567 H australis Australia Canberra Eucalyptus extraction extraction
CANB569566 H australis Canberra extraction extraction
KY081802 X lenis Wu 890714 3 extraction
AG1548 H australis Argentina TierraDelFuego Nothofagus pumilio extraction extraction
AG730 H australis Argentina TierraDelFuego Nothofagus pumilio extraction extraction
AG1872 H australis Argentina TierraDelFuego Nothofagus betuloides extraction extraction
14164MD Huinay extraction
MR11041 H australis Argentina RioNegro Nothofagus dombeyi extraction extraction
14120MD H australis Chile Huinay extraction extraction
PDD 23704
Type
CANB751963 H australis Australia Queensland Eucalyptus extraction extraction
CANB869124 H australis Australia NSW Eucalyptus extraction extraction
CANB869100 H australis Australia NSW Eucalyptus extraction extraction
CANB752088 H australis Australia Queensland extraction extraction
CANB569570 H australis Canberra extraction extraction
CANB752080 H australis Australia Queensland Eucalyptus extraction extraction
CANB569567 H australis Australia Canberra Eucalyptus extraction extraction
CANB569566 H australis Canberra extraction extraction
KY081802 X lenis Wu 890714 3 extraction
AG1548 H australis Argentina TierraDelFuego Nothofagus pumilio extraction extraction
AG730 H australis Argentina TierraDelFuego Nothofagus pumilio extraction extraction
AG1872 H australis Argentina TierraDelFuego Nothofagus betuloides extraction extraction
14164MD Huinay extraction
MR11041 H australis Argentina RioNegro Nothofagus dombeyi extraction extraction
14120MD H australis Chile Huinay extraction extraction
PDD 23705
Type
CANB751963 H australis Australia Queensland Eucalyptus extraction extraction
CANB869124 H australis Australia NSW Eucalyptus extraction extraction
CANB869100 H australis Australia NSW Eucalyptus extraction extraction
CANB752088 H australis Australia Queensland extraction extraction
CANB569570 H australis Canberra extraction extraction
CANB752080 H australis Australia Queensland Eucalyptus extraction extraction
CANB569567 H australis Australia Canberra Eucalyptus extraction extraction
CANB569566 H australis Canberra extraction extraction
KY081802 X lenis Wu 890714 3 extraction
AG1548 H australis Argentina TierraDelFuego Nothofagus pumilio extraction extraction
AG730 H australis Argentina TierraDelFuego Nothofagus pumilio extraction extraction
AG1872 H australis Argentina TierraDelFuego Nothofagus betuloides extraction extraction
14164MD Huinay extraction
MR11041 H australis Argentina RioNegro Nothofagus dombeyi extraction extraction
14120MD H australis Chile Huinay extraction extraction
