# Supplementary material for: Linking morphological and molecular sources to disentangle the case of Xylodon australis
Source: Sci Rep. 2020 Dec 15;10:22004. doi: 10.1038/s41598-020-78399-8 (PMC7738490; doi:10.1038/s41598-020-78399-8)
Supplement: Supplementary file 2 — Supplementary References. [file 41598_2020_78399_MOESM2_ESM.docx]

**Reference List for Supplementary Table S1**

[50] Larsson, K.H. et al. Hymenochaetales: a molecular phylogeny for the hymenochaetoid clade. *Mycologia* **98,** 926–936; 10.3852/mycologia.98.6.926 (2006).

[51] Larsson, K. H., Larsson, E. & Koljalg, U. High phylogenetic diversity among corticioid homobasidiomycetes. *Mycol. Res.* **108,** 983–1002; 10.5941/MYCO.2016.44.4.217 (2004).

[52] Yurchenko, E. & Wu, S. H. Three new species of *Hyphodontia* with peg-like hyphal aggregations. *Mycol. Prog.* **13,** 533–545; 10.1007/s11557-013-0935-1 (2014).

[53] Viner, I., Spirin, V., Zíbarová, L. & Larsson, K. H. Additions to the taxonomy of *Lagarobasidium* and *Xylodon* (Hymenochaetales, Basidiomycota). *MycoKeys* **41,** 65–90; 10.3897/mycokeys.41.28987 (2018).

[54] Rosenthal, L. M. et al. Survey of coriticoid fungi on North American pinaceous forests reveals hyperdiversity, underpopulated sequences databases, and species that are potentially ectomycorrhizal. *Mycologia* **109,** 115–127; 10.1080/00275514.2017.1281677 (2017).

[55] Wang, M. & Cheng, Y. Y. Phylogeny and taxonomy of the genus *Hyphodontia* (Hymenochaetales, Basidiomycota) in China. *Phytotaxa* **309,** 45–54; 10.11646/phytotaxa.309.1.4 (2017).

[56] Chen, C. C., Wu, S. H. & Chen, C. Y. Three new species of *Hyphodontia* s.l. (Basidiomycota) with poroid or raduloid hymenophore. *Mycol. Prog.* **16,** 553–564; 10.10007/s11557-017-1286-0 (2017).

[57] Chen, J. J., Zhou, L. W., Ji, X. H. & Zhao, C. L. *Hyphodontia dimitica* and *H*. *subefibulata* spp. nov. (Schizoporaceae, Hymenochaetales) from southern China based on morphological and molecular characters. *Phytotaxa* **269,** 1–3; 10.11646/pytotaxa.269.1.1 (2016).

[58] Paulus, B., Hallenberg, N., Buchanan, P. K. & Chambers, G. K. A phylogenetic study of the genus *Schizopora* (Basidiomycota) based on ITS DNA sequences. *Mycol. Res.* **104,** 1155–1163; 10.1017/S0953756200002720 (2000).

[59] Jang, Y. et al. Diversity of Wood-inhabiting polyporoid and corticioid fungi in Odaesan National Park, Korea. *Microbiology* **44,** 217–236; 10.5841/MYCO.2016.44.4.217 (2016).

[60] Kan, Y. H., Qin, W. M. & Zhou, L. W. *Hyphodontia mollissima* sp. nov. (Schizoporaceae, Hymenochaetales) from Hainan, southern China. *Mycoscience* **58,** 297–301; 10.1093/bib/bbx108 (2017).

[61] Fukami, T. et al. Assembly history dictates ecosystem functioning: evidence from wood decomposer communities. *Ecol. Lett.* **13,** 675–684; 10.1111/j.1461-0248.2010.01465.x (2010).

[62] Hibbett, D. S. & Binder, M. Evolution of complex fruiting-body morphologies in homobasidiomicetes. *Proc. R. Soc. Lond. B Biol. Sci.* **269,** 1963–1969; 10.1098/rspb.2002.2123 (2002).

[63] Miettinen, O. & Larsson, K. H. *Sidera*, a new genus in Hymenochaetales with poroid and hydnoid species. *Mycol. Prog.* **10,** 131–141; 10.10007/s11557-010-0682-5 (2011).

[64] Zhao, C. L., Cui, B. K. & Dai, Y. C. Morphological and molecular identification of two new species of *Hyphodontia* (Schizoporaceae, Hymenochaetales) from southern China. *Cryptogam. Mycol.* **31,** 87–97; 10.7872/crym.v35.iss1.2014.87 (2014).

[65] Ariyawansa, H. A., Hyde, K. D., Jayasiri, S .C., Buyck, B., Chethana, K. T. & Dai, D. Q. Fungal diversity notes 111–152 – taxonomic and phylogenetic contributions to fungal taxa. *Fungal Divers.* **71**, 27–264; 10.1007/s13225-015-03466-5 (2015).

[66] Parmasto, E. & Hallenberg, N. A. Taxonomic study of phlebioid fungi (Basidiomycota). *Nord. J. Bot.* **20,** 105–118; 10.1111/j.1756-1051.200.tc00740.x (2000).

[67] Kwon, S. L. et al. Identification of three wood decay fungi in Yeoninsan Provincial Park, Korea. *J. Species Res.* **7,** 240–247; 10.12651/JSR.2018.7.3.240 (2018).

[68] Brazze, N. J. et al. Disturbance and diversity of wood-inhabiting fungi: effects of canopy gaps and downed woody debris. *Biodivers. Conserv.* **23,** 2155–2172; 10.1007/s10531-014-0710-x (2014).

[69] Cochrane, G. et al. Facing growth in the European Nucleotide Archive. *Nucleic Acid Res.* **41,** 30–35; 10.1093/nar/gks1175 (2013).
